# GAPDH heme delivery to Indoleamine 2,3-dioxygenase 1 involves their complex formation and complementary charge pairing at the protein-protein interface

**DOI:** 10.1016/j.jbc.2025.110443

**Published:** 2025-07-01

**Authors:** Pranjal Biswas, Yue Dai, Dhanya T. Jayaram, Priya Das Sinha, Saurav Misra, Jesus Tejero, Belinda Willard, Dennis J. Stuehr

**Affiliations:** 1 Department of Inflammation and Immunity, Cleveland Clinic Lerner College of Medicine of Case Western Reserve University School of Medicine, Cleveland, Ohio, USA; 2 Department of Biochemistry and Molecular Biophysics, Kansas State University, Manhattan, Kansas, USA; 3 Heart, Lung, Blood and Vascular Medicine Institute, University of Pittsburgh, Pittsburgh, Pennsylvania, USA; 4 Department of Pharmacology and Chemical Biology, University of Pittsburgh, Pittsburgh, Pennsylvania, USA; 5 Proteomics and Metabolomics Core, Cleveland Clinic Lerner College of Medicine of Case Western Reserve University School of Medicine, Cleveland, Ohio, USA

**Keywords:** GAPDH, IDO1, heme insertion, mass spectrometry, protein-protein interaction

## Abstract

In eukaryotes, the last steps of heme biosynthesis occur in mitochondria and so heme must be transported to reach many heme-dependent proteins that mature and function outside this organelle. Although the enzyme glyceraldehyde 3-phosphate dehydrogenase (GAPDH) has emerged as a key intracellular heme chaperone, how it performs heme deliveries to its numerous clients is poorly understood. It is unknown if handoffs of the GAPDH-bound heme require that it make direct contact with its clients or instead involve GAPDH passing its heme to middlemen proteins to execute the final heme transfers. To address this question, we studied GAPDH heme transfer to the client protein indoleamine 2,3-dioxygenase 1 (IDO1), whose enzyme activity is heme-dependent and regulates mammalian immune responses and cancer progression. A chemical crosslinking-mass spectrometry approach identified two Lys residues that formed an inter-protein crosslink across a previously uncharacterized GAPDH-IDO1 interface. This guided our building a model of the GAPDH-IDO1 complex so we could interrogate by point mutagenesis the role of the GAPDH-IDO1 contact in enabling delivery of GAPDH heme to IDO1. We characterized behaviors of the GAPDH and IDO1 variants in their purified form and when they were expressed in the HEK293T human cell line. This revealed GAPDH heme transfer to IDO1 in cells requires that they make a direct contact which relies on a specific Lys-Asp charge pairing interaction forming across the complex interface. These findings illuminate a key step in the maturation of functional IDO1 and improve our understanding of how GAPDH may perform its heme trafficking function in mammals.

Many life processes depend on proteins that bind a special form of iron called iron-protoporphyrin IX (heme) (1, 2, 3). In eukaryotes, the last steps of heme biosynthesis occur in mitochondria and thus heme must be transported to reach the many heme-dependent proteins that function outside this organelle (4). Typically, such proteins are initially heme-free after their biosynthesis and must acquire heme to become functional. Remarkably, the ubiquitous glycolytic enzyme glyceraldehyde 3-phosphate dehydrogenase (GAPDH) has emerged as an important intracellular chaperone of mitochondrially generated heme in eukaryotes (5). Indeed, heme deliveries to a wide variety of hemeproteins in mammalian cells including NO synthases, soluble guanylyl cyclase (sGC), cytochrome P450s, myoglobin, hemoglobins, indoleamine dioxygenase 1 (IDO1), and tryptophan dioxygenase (TDO) have been shown to depend on GAPDH and its specific heme-binding ability (6, 7, 8, 9, 10, 11). While GAPDH clearly plays a role in cell heme allocation *via* its heme binding function, exactly how the GAPDH heme is delivered to its clients is unknown. One key question concerns the final heme “handoff” to the hemeprotein recipients: Does this step depend on GAPDH making direct contact with the client protein, or does it instead involve heme handoff from GAPDH to unidentified middlemen proteins, which would then execute the final heme transfers? Co-immunoprecipitation studies have shown that GAPDH associates with all of its known heme protein clients in cells (6, 8, 9, 10, 11), and studies with purified GAPDH and its client proteins myoglobin, sGC, IDO1, and TDO show that GAPDH forms a direct complex with their apo forms at μM concentrations and that the affinity is diminished when the client proteins contain bound heme (8, 9, 12). Although this indicates that GAPDH can associate with its apoprotein clients to form direct complexes, it does not answer whether such direct GAPDH-client contact is involved in or required for the heme deliveries.

To address this question, we sought to identify amino acids located at the interface of a GAPDH–client protein complex, reasoning that this would enable us to test their importance in stabilizing the GAPDH-client interaction and in turn to determine whether direct GAPDH-client contact mediates the GAPDH heme delivery. We are investigating this question by applying a chemical crosslinking-mass spectrometry approach (13) that utilizes a mass-spec-cleavable, Lys-targeted crosslinker to identify Lys residues located in the GAPDH-client protein interface that are close enough to form intermolecular protein-protein crosslinks. Here, we utilize this approach to interrogate the complex formed between GAPDH and its client hemeprotein IDO1. Cellular heme delivery to IDO1 depends on GAPDH heme binding ability (9) and IDO1 catalytic activity is directly related to its heme content (9). IDO1 catalyzes conversion of L-Trp to L-Kynurenine (L-Kyn) (14), whose biological roles include resolution of the immune response (15) and promotion of tumor escape from immune surveillance (16). We identified two Lys residues that form an interprotein crosslink across the previously uncharacterized GAPDH-IDO1 interface. Using the available protein crystal structures of GAPDH and IDO1 (17, 18), we generated computer-based models of the GAPDH-IDO1 complex. This allowed us to further interrogate the interface residues in enabling complex formation and ultimately to determine the importance of direct contact between GAPDH and IDO1 for GAPDH-dependent heme allocation to IDO1 in cells. Our findings illuminate the mechanism of this step in the maturation of functional IDO1 and helps to advance general understanding of how GAPDH heme trafficking takes place in mammalian cells.

## Results

### GAPDH can transfer heme to apo-IDO1 *in vitro*

Previously, we found that purified forms of human GAPDH and apo-IDO1 form a complex at low μM concentrations (9). To test the functional significance, we investigated heme transfer from a pre-formed GAPDH heme complex upon mixing it with apo-IDO1 and incubating at 37 °C for 30 min (the apo-IDO1 preparation used here contained some residual heme; 0.1 mol/mol). We also utilized a human recombinant GAPDH that contains a tetra-Cys insertion sequence near its heme binding site (TC-GAPDH) which allows it to specifically bind the FlAsH reagent and thereby report on the level of its bound heme due to the heme quenching its FlAsH fluorescence emission intensity (19). After the 30-min reaction, the solution was concentrated by filtration and injected onto an analytical gel filtration column to separate the TC-GAPDH and IDO1 proteins. A comparison of the UV-vis spectra recorded for the TC-GAPDH-heme complex and IDO1 samples both prior to and after their reaction and separation indicate that the heme content of the TC-GAPDH became diminished by about 50% (from 0.4 mol of heme per tetramer initially) and this corresponded to an increase in the heme content of IDO1 (Fig. S1*A*). This was accompanied by a doubling of the IDO1 catalytic activity (Fig. S1*B*), indicating that transfer of the heme into the apo-IDO1 created a catalytically functional enzyme. In a second approach, we FlAsH-labeled the TC-GAPDH-heme complex and followed the kinetics of its heme loss after mixing it with the apo-IDO1 preparation. The traces in Fig. S1*C* show that binding heme into FlAsH-TC-GAPDH decreased its fluorescence by 50% and that in the absence of added apo-IDO1, its bound heme content remained stable over the 30-min incubation period. However, in samples where apo-IDO1 was added at time = 0 we observed a gradual loss of heme from the FlAsH-TC-GAPDH as indicated by the gradual fluorescence increase with time, consistent with heme transfer from FlAsH-TC-GAPDH into apo-IDO1. Thus, the complex formed between purified GAPDH and apo-IDO1 engaged in heme transfer and generated a catalytically functional IDO1.

### MS-cleavable crosslinking identifies a Lys-Lys crosslink in the GAPDH-apo-IDO1 protein-protein interface

We next deployed a chemical crosslinking-mass spectrometry approach to identify Lys residues in the complex formed between purified GAPDH and apo-IDO1 that may be located close enough across the protein-protein interface to undergo crosslinking with disuccinimidyl sulfoxide (DSSO) (13, 20). The DSSO crosslinker contains two Lys-reactive N-hydroxy-succinimide (NHS) ester groups separated by an MS-cleavable sulfoxide linker that is 10.1 Å long (Fig. S2). Subsequent MS analysis of the peptides generated after protease digestion of the DSSO-reacted protein complex identified many intra-protein Lys-Lys crosslinks within GAPDH or apo-IDO1, as expected (Tables S1–S3), and also identified three inter-protein crosslinks formed between GAPDH and apo-IDO1 (Table S2). This revealed that these lysines are positioned very near each other (*i.e.*, within about 10 Å) at the interface of the complex formed by purified GAPDH and apo-IDO1.

### K101 in IDO1 enables its interaction with GAPDH and its heme procurement

**Figure 1:**
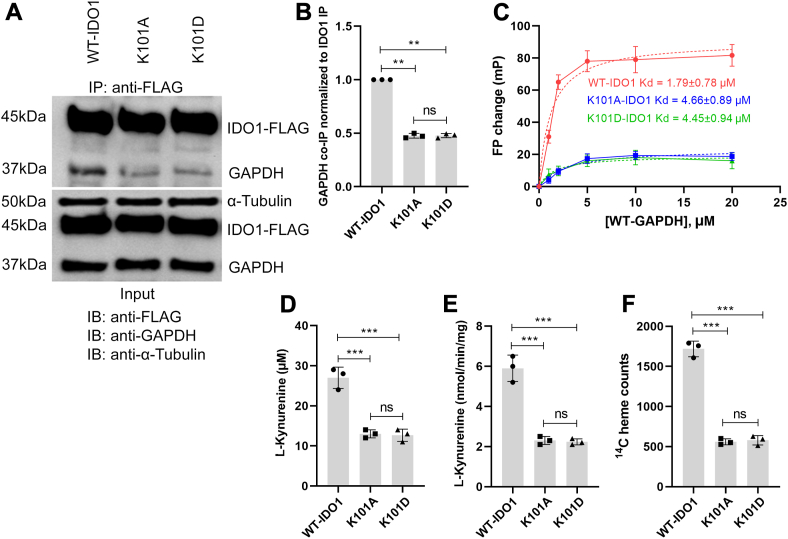
**K101 in IDO1 enables its interaction with GAPDH and its heme procurement.** FLAG-tagged wild type IDO1 or the K101A and K101D IDO1 variants were each transiently expressed in HEK293T cells for 48 h and then cell supernatants were prepared. Each IDO1 protein was also recombinantly expressed in *E.coli* and then purified. The HEK293T cell supernatants (equal protein) were immunoprecipitated with an anti-FLAG antibody followed by SDS-PAGE and Western blot analysis with anti-GAPDH and anti-FLAG antibodies. *Panel A*, representative Western blot comparing amounts of GAPDH with each IDO1 IP sample along with the input levels of the proteins and of α-tubulin. *Panel B*, densitometric quantification of the bound GAPDH, values reported relative to the wild-type IDO1 sample, mean ± S.D, n = 3. *Panel C*, change in residual fluorescence polarization signal upon titrating each indicated fluorescently labeled IDO1 protein (100 nM) with GAPDH. Points are mean ± SD, n = 3. Single exponential lines of best fit are shown as dashed lines and the derived Kd values are noted. *Panels D* and *E*, IDO1 activity measured by L-Kyn buildup in the cell culture fluid or by L-Kyn generation by the cell supernatants, respectively, for cells expressing the indicated IDO1 proteins. Points are mean ± SD, n = 3. *Panel F*, relative heme contents of the indicated IDO1 proteins expressed in cells that had been given the heme precursor ^14^C-δ-ALA. Bars are the mean ± SD, n = 3. ∗∗∗*p* < 0.001, ∗∗*p* < 0.01; ns, not significant.

To probe the functional importance of the cross-links between Lys (K101 or K397 in IDO1 and K334 or K215 in GAPDH), we utilized targeted mutagenesis to change each Lys to a neutral or negatively charged amino acid. We transiently expressed each variant in a human cell line (HEK293T cells), and recombinantly expressed and purified the variants in *E.* *coli* to compare their behaviors compared to wild-type GAPDH and IDO1. K397A mutation in IDO1 did not affect its activity or heme levels (data not shown) so we focused on the K101 mutations in IDO1 to move forward. Expression levels of FLAG-tagged K101A and K101D IDO1 variants in the transfected HEK293T cells were similar to that of FLAG-wild type IDO1, and the expression of these proteins did not alter the cell GAPDH levels (Fig. S4, *A*–*C*). Co-immunoprecipitation experiments using an anti-FLAG Ab showed that the amount of GAPDH immunoprecipitated with the IDO1 K101A or D variants was about 50% less than that with wild-type FLAG-IDO1 (Fig. 1, *A* and *B*). Likewise, the purified K101A and K101D IDO1 variants exhibited lower binding affinity toward purified GAPDH relative to wild-type apo-IDO1, as judged from their lower increases in residual fluorescence polarization upon titration with purified GAPDH relative to the titration of an equivalent concentration of wild-type purified IDO1 protein (Fig. 1*C*). These data suggest that K101 helps to mediate the IDO1 interaction with GAPDH both when they are studied in their purified forms or expressed together in HEK293T cells.

We next tested how the K101A and K101D substitutions would impact IDO1 heme procurement in HEK293T cells. First, we measured their heme-dependent catalytic activities (L-Kyn production from L-Trp) relative to wild-type IDO1. Cells that expressed the K101A or K101D IDO1 had about 60% less L-Kyn buildup in their culture medium over a 5 h period (Fig. 1*D*). Likewise, supernatants from these cells exhibited catalytic activities that were reduced by 65% relative to wild type (Fig. 1*E*). To measure heme incorporation in the IDO1 variants, we added the heme precursor delta-aminolevulinic acid (^14^C-δ-ALA) to HEK293T cells to allow mitochondrial generation of ^14^C-heme (5). ^14^C heme counts in the IDO1 immuno-precipitants showed that the lower catalytic activities of the IDO1 K101A and D variants were associated with a 68% decrease in their heme contents relative to wild-type IDO1 (Fig. 1*F*). When cell supernatants containing wild-type IDO1 or either variant were titrated with exogenous heme, the added heme increased their catalytic activities to similar extents (Fig. S5), confirming that incorporating the K101A or D substitutions did not alter the inherent heme binding affinity or catalytic activity of the IDO1 protein. Thus, the IDO1 K101A and K101D variants exhibited a diminished interaction with GAPDH *in* *vitro* and in cells, and this correlated with their having a reduced heme content and a diminished catalytic L-Kyn production.

We similarly investigated the behavior of the GAPDH K334A and K215A variants in the HEK293T cells by employing our previously established siRNA knockdown and rescue strategy (21). We knocked down native GAPDH expression in HEK293T cells using a targeted siRNA approach, and then transiently transfected the cells with an IDO1 expression vector along with either an empty vector or vectors designed to express siRNA-resistant forms of HA-tagged wild-type GAPDH or GAPDH K334A variant. When wild-type or K334A GAPDH were expressed in these cells, they behaved identically with respect to their expression levels, interactions with IDO1, and IDO1 L-Kyn production (Fig. S6, *A*–*G*). This indicated that a loss of charge at K334 in GAPDH, on its own, did not significantly alter GAPDH interaction with IDO1 or diminish GAPDH-dependent heme delivery to IDO1. For the K215A mutation in GAPDH, we observed similar results to the K334A mutant; its mutation did not significantly alter GAPDH interaction with IDO1 or diminish GAPDH-dependent heme delivery to IDO1 (data not shown).

### A structural model of the GAPDH-IDO1 complex identifies potential charge pairing partners for K101

**Figure 2:**
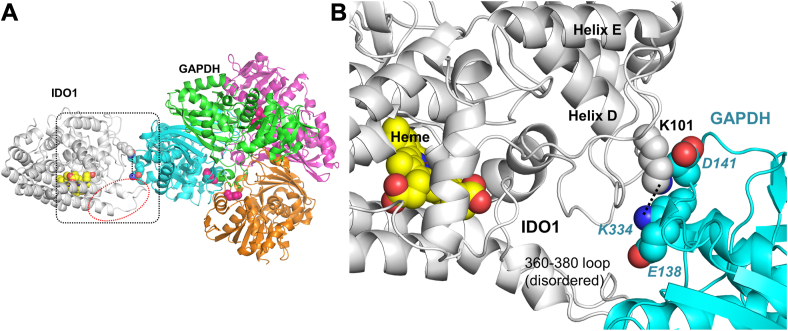
**A structural model of the GAPDH-IDO1 complex identifies potential charge pairing partners for K101.** GAPDH-IDO1 model complexes were computer-generated using the available protein crystal structures along with ColabFold and HADDOCK software. *Panel A*, a representative model complex. The IDO1 (*gray*) and GAPDH tetramer (*multi-colored*) proteins are shown primarily as ribbon structures with some highlighted regions or residues shown in space filling designation. Highlights: IDO1 heme cofactor (*yellow* with *red* propionate carboxylates), Crosslinked Lys residues (*blue*) and nearby Asp or Glu residues in GAPDH (*red*), IDO1 J-K loop (residues 360–380, *dashed red oval*), and the heme-binding His residues in GAPDH (*magenta*). *Panel B*, closeup of the model region outlined by the *dashed black* square in *panel A*, with crosslinked Lys and nearby Asp and Glu residues identified as marked.

Given that IDO1 K101 helped enable IDO1-GAPDH complex formation and IDO1 heme acquisition, we wondered if it might act by it charge pairing across the interface with negatively charged residues near K334 on the GAPDH surface. Using crystal structures of GAPDH and IDO1 as guides (17, 18), we carried out structural modeling of their complex. Because a segment of IDO1 called the J-K loop forms part of the opening to its heme-binding site and is partly disordered in the reference IDO1 crystal structure, we used ColabFold (22) to generate a model of a full IDO1 structure (with heme bound). We then used HADDOCK (23) to generate models of the GAPDH-IDO1 complex, specifying a strong distance constraint between IDO1 K101 and GAPDH K334 to satisfy the experimentally observed crosslink. While HADDOCK generated a range of configurations for the complex, the resulting models consistently placed an IDO1 loop that contains K101 near a short, highly surface-exposed helix of GAPDH that contains acidic residues E138 and D141 very near the crosslinking GAPDH K334 residue (Fig. 2, *A* and *B*). We posited that the IDO1 K101 residue might interact by complementary charge pairing with either or both of these GAPDH acidic residues. Notably, residue identity and/or charge at K101, K334, E138, and D141 are conserved among various organisms that express both IDO1 and GAPDH (Fig. S7, *A* and *B*).

### Charge reversal at D141 and/or E138 in GAPDH does not affect its ability to obtain or carry heme

Based on the modeling results, we next constructed in GAPDH the single charge reversal variants E138R or D141R and the double charge reversal variant (E138R + D141R). To determine whether these mutations impact GAPDH heme procurement from the cell mitochondria, which, if observed, would be a complicating factor for our study, we created the variants in both our normal GAPDH construct and in our HA-TC-GAPDH reporter construct. The HA-TC-GAPDH construct, upon binding a FlAsH reagent (24), fluoresces and thus indicates its heme binding by a loss of its FlAsH fluorescence (19). We expressed each of the three FlAsH-labeled HA-TC-GAPDH variants in HEK293T cells and then administered δ-ALA/Fe to the cells to boost their mitochondrial heme biosynthesis (19). We then determined the abilities of the variants to bind the mitochondria-generated heme, as indicated by a loss in their FlAsH fluorescence. We also measured the intrinsic heme affinities of each FlAsH-HA-TC-GAPDH variant protein by titrating heme into the supernatants prepared from cells that expressed each variant. All three HA-TC-GAPDH variants were similar to wild-type HA-TC-GAPDH in their kinetics and extent of mitochondrial heme procurement following the δ-ALA/Fe addition to the cells (Fig. S8, *A–D*), and they also displayed identical heme affinities upon heme titration of the cell supernatants (Fig. S9, *A–E*). Thus, we conclude the E138R and D141R substitutions did not significantly impact the ability of GAPDH to obtain or bind heme, which are the essential upstream steps needed for GAPDH-dependent heme allocation to IDO1 in cells.

### Charge reversal at D141 in GAPDH diminishes its interaction with and heme allocation to IDO1

We next employed our siRNA GAPDH knockdown and rescue strategy (described above) to examine whether the E138R, D141R, or the double charge reversal (E138R + D141R) GAPDH variants interact with IDO1 in cells (9, 21). We knocked down native GAPDH in HEK293T and transiently transfected them to express IDO1 along with either empty vector or vectors that express siRNA-resistant HA-tagged wild-type GAPDH or each of the three GAPDH variants. We examined the extent of GAPDH knockdown, expression levels of each HA-GAPDH protein and IDO1, HA-GAPDH variant association with IDO1 by co-IP, and IDO1-mediated L-Kyn production.

**Figure 3:**
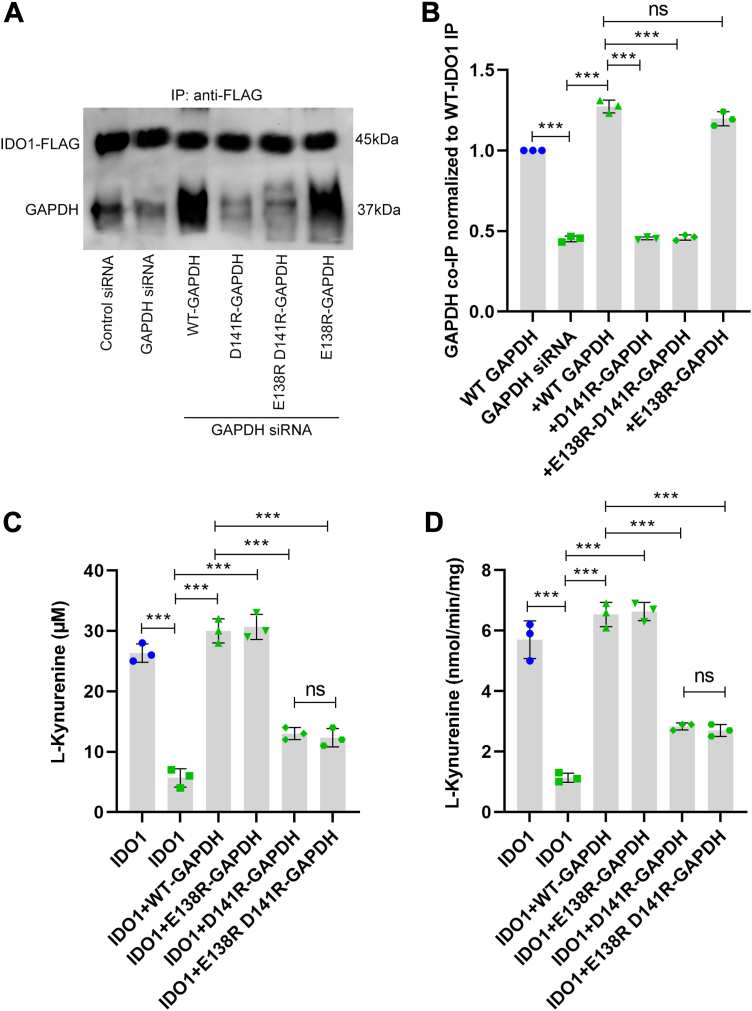
**Charge reversal at D141 in GAPDH diminishes its IDO1 interaction and its heme allocation to IDO1.** GAPDH expression either was not (*blue dots*) or was (*green triangles*) knocked down in HEK293T cells by siRNA treatment, and then the cells were transiently transfected to express FLAG-IDO1 along with either empty vector or vectors designed to express siRNA-resistant wild-type HA-GAPDH or each indicated GAPDH variant. *Panel A*, HEK293T cell supernatants (equal protein) underwent IP with an anti-FLAG antibody followed by SDS-PAGE and Western blot analysis. The representative Western blot compares amounts of GAPDH and FLAG-IDO1 in the IP samples. *Panel B*, densitometric quantification of the bound GAPDH relative to the FLAG-IDO1 band in the IP samples, mean ± S.D, n = 3. *Panels C* and *D*, IDO1 activity measured by L-Kyn buildup in the cell culture fluid or by L-Kyn generation by the cell supernatants, respectively. Bars are mean ± SD, n = 3. ∗∗∗*p* < 0.001; ns, not significant.

In HEK293T cells that expressed IDO1, the GAPDH-targeted siRNA treatment lowered GAPDH expression by approximately 60% relative to the scrambled siRNA control, and transfecting the knocked-down cells to re-express either wild-type GAPDH or each GAPDH variant recovered the total cell GAPDH protein expression level without altering cell IDO1 expression (Fig. S10, *A*–*C*). In Co-IP experiments with the cell supernatants using an IDO1 antibody, we observed decreased association of the D141R GAPDH and the E138R + D141R GAPDH variants with IDO1 relative to wild-type HA-GAPDH or the E138R GAPDH variant (Figs. 3, *A* and *B*, and S11). We observed similar binding differences when we examined the association between the purified forms of the IDO1 and GAPDH proteins using fluorescence polarization (Fig. S12). These differences in GAPDH-IDO1 protein interaction were mirrored by differences in IDO1 activity: knockdown of GAPDH decreased L-Kyn production and the IDO1 activity of cell supernatant by 50 to 60% (Fig. 3, *C* and *D*). These losses were rescued by re-expression of wild-type GAPDH or the E138R GAPDH variant, but not by re-expression of the D141R or the E138R + D141R GAPDH variants (Fig. 3, *C* and *D*). Thus, as observed for K101 in IDO1, the D141 residue in GAPDH also enabled GAPDH-IDO1 complex formation in cells, and their degree of complex formation directly correlated with the degree of heme-dependent IDO1 activity in the cells.

### A combined charge reversal at IDO1 K101 and GAPDH D141 rescues their complex formation and IDO1 activity

We next investigated whether charge-pairing between IDO1 K101 and GAPDH D141 across the protein–protein interface is what enables the IDO1-GAPDH interaction needed for IDO1 heme procurement and enzyme activity. As noted earlier, we already found that the expression of K101D IDO1 and D141R GAPDH variants on their own resulted in poor GAPDH-IDO1 association and poor heme allocation to IDO1 in cells containing the respective GAPDH or IDO1 wild-type partners. We reasoned that if these two variants were expressed in combination (*i.e.*, K101D in IDO1 and D141R in GAPDH) it might enable formation of a complementary charge interaction of reversed polarity across the protein-protein interface and thus enable normal GAPDH-IDO1 interaction and IDO1 heme allocation if these aspects do in fact depend on a charge pairing interaction at this site.

**Figure 4:**
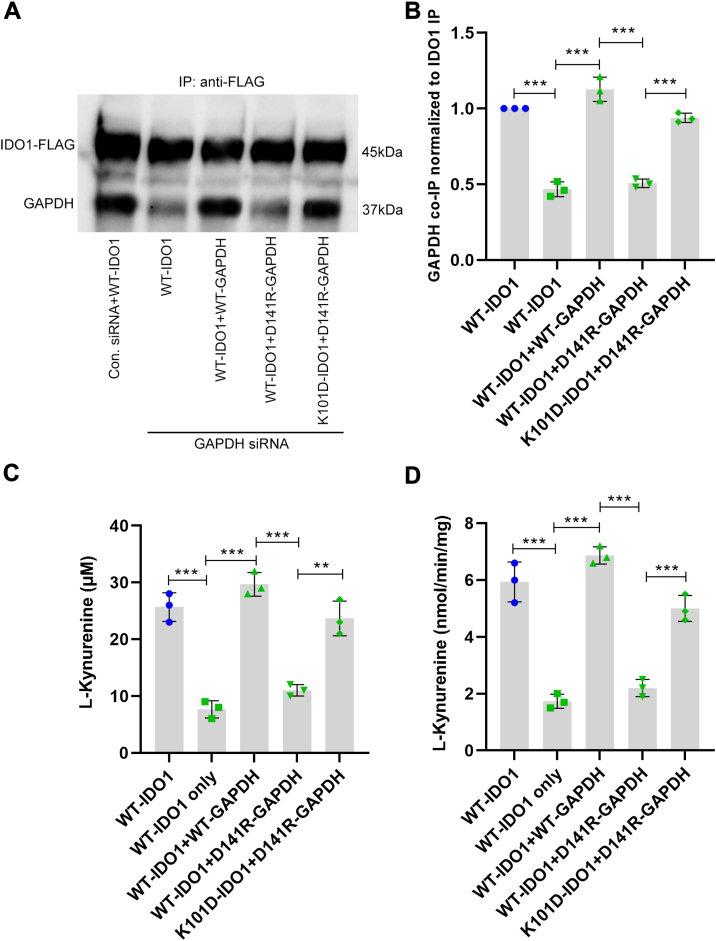
**Combined charge reversals at IDO1 K101 and at GAPDH D141 rescues their complex formation and IDO1 activity.** GAPDH expression either was not (*blue dots)* or was (*green triangles*) knocked down in HEK293T cells by siRNA treatment, and then the cells were transiently transfected to express FLAG-IDO1 wild type or the K101D variant along with either empty vector or vectors designed to express siRNA-resistant wild-type HA-GAPDH or the D141R variant. *Panel A*, HEK293T cell supernatants (equal protein) underwent IP with an anti-FLAG antibody followed by SDS-PAGE and Western blot analysis. The representative Western blot compares the amounts of GAPDH and FLAG-IDO1 in the IP samples. *Panel B*, densitometric quantification of the bound GAPDH relative to the FLAG-IDO1 bands in the IP samples, mean ± S.D, n = 3. *Panels C* and *D*, IDO1 activity measured by L-Kyn buildup in the cell culture fluid or by L-Kyn generation by the cell supernatants, respectively. Bars are mean ± SD, n = 3. ∗∗∗*p* < 0.001; ns, not significant.

GAPDH-directed siRNA treatment of HEK293T cells lowered their GAPDH expression level by approximately 60%, which was rescued by re-expression of wild type or D141R GAPDH, without affecting co-expression of wild-type IDO1 or the K101D IDO1 variant (Fig. S13, *A* and *B*). In Co-IP experiments with anti-IDO1 (Figs. 4, *A* and *B* and S13*C*), the amount of GAPDH associated with the expressed wild-type IDO1 decreased in the GAPDH knockdown cells as expected. Expression of wild-type GAPDH in the knockdown cells restored the level of GAPDH association with wild-type IDO1, whereas the expression of the D141R HA-GAPDH variant did not. However, in the knockdown cells that expressed the IDO1 K101D variant, expression of the D141R HA-GAPDH did restore a normal level of association (Fig. 4, *A* and *B*). These differences in the levels of GAPDH-IDO1 protein associations were mirrored by differences in the IDO1 activities as measured by L-Kyn accumulation in cell cultures (Fig. 4*C*) and by the IDO1 activities measured for the cell supernatants (Fig. 4*D*). These results confirm that a charge pairing interaction across the interface of the GAPDH-IDO1 complex at positions 141 in GAPDH and 101 in IDO1 is critical for enabling the GAPDH-IDO1 interaction and heme transfer.

### The influence of GAPDH D141 is client protein-specific

To examine whether the effect of GAPDH D141 on the GAPDH-IDO1 interaction and heme delivery is specific, we repeated our experiments in HEK293T cells that were expressing the hemeprotein TDO in place of IDO1. TDO is dissimilar to IDO1 in its primary sequence and structure (25), but like IDO1, it catalyzes L-Trp oxidation to L-Kyn and also interacts with and depends on GAPDH to receive mitochondrially-generated heme in cells (9). Unlike their effects on IDO1, we found that all the GAPDH variants (including D141R) interacted with TDO in the cells or in purified forms similar to wild-type GAPDH (Fig. S14, *A*–*E*). Moreover, all the GAPDH variants supported an equivalent TDO heme allocation as measured by L-Kyn production or by TDO activity in cell supernatants. Thus, D141 was specifically important for GAPDH interaction with and heme transfer to IDO1 by this comparison.

## Discussion

Although many different heme proteins rely on GAPDH for their heme allocations in cells, how the GAPDH-dependent heme transfers occur is largely unclear. Our study reveals that (i) a complex formed by purified GAPDH and apo-IDO1 is competent for heme transfer from GAPDH into the apo-IDO1, and (ii) GAPDH-dependent heme delivery to IDO1 in cells requires that they directly associate with one another as enabled by a specific charge pairing interaction that forms across their protein-protein interface. These findings shed new light on how IDO1 matures to its catalytically functional form and provide a first example of how GAPDH may participate in heme deliveries to its client proteins in mammalian cells.

### A direct charge-pairing interaction across the GAPDH-IDO1 interface is critical for effective heme transfer

Our study first identified Lys residues in the GAPDH-apo-IDO1 complex that could undergo crosslinking across the protein–protein interface. This provided a geometric restraint to build a docked structural model of the GAPDH-IDO1 complex using the available protein crystal structures, which in turn allowed us to probe what amino acid residues might enable their direct contact and if direct GAPDH contact was important for heme delivery to apo-IDO1 in cells. Because this required that we generate point substitutions that were involved or were located near the crosslinking Lys residues in either protein, it was imperative to first determine if the point substitutions would impact other characteristics apart from their interaction that determine their heme binding abilities and in the case of IDO1 its heme-dependent catalysis. We confirmed that none of the IDO1 substitutions impacted its expression level, intrinsic heme-binding affinity, or intrinsic heme-dependent catalytic activity (L-Kyn production) compared to wild-type IDO1. Likewise, none of our GAPDH substitutions altered its expression level in cells, its ability to obtain mitochondrially-generated heme, or its intrinsic heme binding affinity, all of which are important upstream features that enable intracellular GAPDH to participate in heme deliveries (9, 26). Thus, based on our previous finding that GAPDH binds preferentially with apo-IDO1 *versus* heme-replete IDO1 both in purified and in cellular systems (9), we could confidently discern any negative impacts that the point substitutions might have on the IDO1 heme content in cells were due to a disruption of the GAPDH-apo-IDO1 complex formation. This in turn allowed us to discern whether heme delivery to IDO1 depended on direct contact with GAPDH in cells. Our results showed that this is indeed the case: We found that IDO1 heme acquisition as measured by ^14^C heme incorporation or by heme-dependent catalytic activity (L-Kyn production) was directly proportional to the level of GAPDH-apo-IDO1 complex formation in cells. Moreover, we found that the GAPDH-dependent heme transfer to apo-IDO1 and their protein-protein interaction heavily relied on two oppositely charged surface residues that our model of the GAPDH-IDO1 complex predicted to be located very close to one another across the protein-protein interface, namely K101 in IDO1 and D141 in GAPDH. Mechanistically, the importance of these residues appeared due to their engaging in a complementary charge pairing interaction across the interface. This was validated by our finding that pairing the GAPDH and IDO1 variants that possessed reversed charges at these two positions (and thus could restore a charge paring interaction of reversed polarity) supported normal levels of GAPDH-apo-IDO1 interaction and IDO1 heme content in the cells, thereby rescuing the diminished levels that were supported by either variant when they were individually expressed in combination with their respective wild-type partner proteins. Overall, this suggests that GAPDH heme delivery to apo-IDO1 relies on their direct complex formation as enabled by a specific surface charge-pairing interaction between the two proteins.

### Potential GAPDH-IDO1 heme transfer pathways

**Figure 5:**
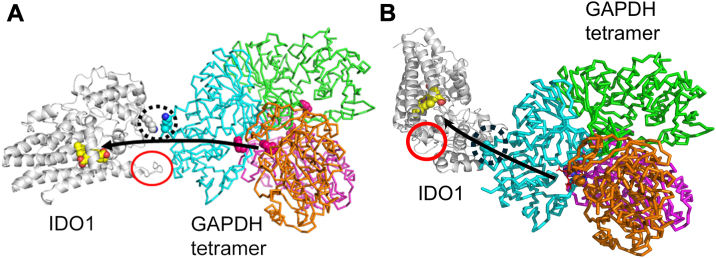
**Representative IDO1-GAPDH model complexes highlighting potential heme transfer pathways.** GAPDH-IDO1 model complexes were computer-generated using the available protein crystal structures along with ColabFold and HADDOCK software (*A*) or by Chai-1 (*B*). The IDO1 (*gray*) and GAPDH tetramer (*multi-colored*) proteins are shown primarily as ribbon structures with some highlighted regions or residues shown in space filling designation. Highlights: IDO1 heme cofactor (*yellow* with *red* propionate carboxylates), Crosslinked Lys residues (space filling residues in *dashed black ovals*), IDO1 J-K loop (*red ovals*), GAPDH heme-binding His residues (*pink* or *copper colored balls*), and possible heme transfer pathways (*black arrows*).

Although we found that GAPDH heme allocation to apo-IDO1 depends on direct interaction between these proteins in cells, and that heme can transfer from GAPDH to apo-IDO1 when the two purified proteins are in complex, the exact heme transfer pathway from GAPDH to apo-IDO1 is not yet clear. We therefore modeled structures of IDO1-GAPDH complexes to examine potential paths for direct heme transfer. The GAPDH tetramer is thought to contain two equivalent heme-binding sites in clefts that form between two protomers on each side of the tetramer (5). HADDOCK-based modeling generated a range of IDO1-GAPDH complexes, which overall allow for a shielded or semi-shielded heme transfer pathway from a GAPDH heme-binding cleft to the opening of the IDO1 heme-binding pocket (Figs. 5*A* and S15, *A–F*). In these, the IDO1–K101 interaction with a short surface-exposed GAPDH helix that contains K334 and D141 (Figs. 5*A* and S15 black ovals) provides the critical bridging point to potentiate inter-protein heme transfer. The range of modeled complexes reveals only limited or no other interactions between the two proteins. The interaction between K101 in IDO1 and D141 in GAPDH restricts IDO1 to interact primarily with one of the four protomers in the GAPDH tetramer, such that one of the two heme-binding clefts of the tetramer is closer to IDO1 and poised to release heme toward IDO1. In these models, a semi-shielded 50 to 60 Å potential heme-transfer path is formed (Figs. 5*A* and S15, black arrows), starting from the GAPDH heme-binding His residues in the cleft (Figs. 5*A* and S15, magenta balls), continuing along the IDO1-interacting GAPDH protomer and then along the IDO1-GAPDH interaction site into the mouth of the IDO1 heme pocket.

We also utilized Chai-1-based protein-protein complex calculations in place of HADDOCK to generate IDO1-GAPDH complex models (Figs. 5*B* and S16, *A* and *B*). Overall, the Chai-1 outputs were distributed along a narrower range of relative orientations between the proteins than those generated using HADDOCK. In these orientations, IDO1 was at a slightly more “open” angle relative to the interacting GAPDH protomer. However, similar heme-site to heme-site distances are present in both the Chai-1 and HADDOCK outputs, and the putative heme transfer path is also semi-shielded by the same elements in the GAPDH protomer and IDO1 in both series of output models.

In the final part of the possible heme transfer pathway, the IDO1 J-K loop (Figs. 5, S15, and S16, red ovals) is typically positioned to provide additional shielding for the heme. Transient interactions and packing of the heme against the protein elements that form the transfer pathway would be critical to avoid heme diffusion away from the GAPDH-IDO1 complex. In a smaller fraction of the HADDOCK-generated structures, the relative orientation of IDO1 and GAPDH allows IDO1 to make additional contacts to an adjacent GAPDH protomer (Fig. S15*E*). Although these orientations possess a notably shorter (∼30 Å) and more buried heme transfer pathway, the IDO1 J-K loop, as configured in our ColabFold model, comes close to clashing with GAPDH (Fig. S15*E*, red oval) and partly blocks the ostensible heme transfer pathway. It is conceivable that the J-K loop would adopt different conformations to allow the heme to move past into the IDO1 heme pocket, because the J-K loop is disordered or poorly defined in most IDO1 crystal structures (17) and is expected to be flexible. Overall, the models generated using both HADDOCK and Chai-1 are consistent with the formation of a dynamic complex between IDO1 and GAPDH, where the two proteins may adopt a range of relative orientations. Most of these orientations are compatible with a heme transfer occurring between the two interacting proteins in which the protein structures partly or substantially shield the heme pathway from solvent. Future efforts to determine the heme transfer pathway and whether it directly proceeds between the two proteins or *via* another route are underway.

### Biological relevance

The heme level in IDO1 directly determines its production of L-Trp metabolites that govern the immune response and other processes, including blood pressure regulation, emotional state, and cancer progression (27, 28, 29, 30, 31, 32). Understanding the process of heme delivery to IDO1 is, therefore, broadly important and provides insights into new ways to manipulate IDO1 activity for therapeutic benefit. It is noteworthy that IDO1 is natively and even predominantly in its heme-deficient form in cells and tissues (33, 34). We recently showed that biological levels of NO either up- or downregulate the GAPDH-dependent heme delivery into IDO1, depending on the level of the NO exposure (19, 21). IDO1 activity is also known to decrease in cells in which heme oxygenase 1 or NO synthase activities are increased (35, 36, 37). Overall, these studies imply that IDO1 activity is subject to dynamic physiological changes in its heme level. How the GAPDH-IDO1 interaction participates in enabling these changes can now be investigated.

## Experimental procedures

### Expression and purification of IDO1

N-terminally His6-tagged human IDO1 (IDO1) was cloned in pHis-parallel1 vector and purified with minor modifications for His tagged proteins as reported previously (9). Briefly, *Escherichia coli* BL21 (DE3) log phase culture in Terrific Broth was used to express IDO1 protein upon induction with 1 mM IPTG at RT for 48h. The protein was purified using Ni-NTA resin (Thermo) and its purity checked by SDS-PAGE and Coomassie staining. During the purification of His6-IDO1, heme was not added to the bacterial growth medium or during protein induction with IPTG. The heme bound in purified IDO1 was found to be around ten percent per mole of the total purified protein, as measured by a heme chromogen assay (38). No effort was made to remove this residual heme.

### Purification of WT and mutant TC-GAPDH proteins from bacteria

GST-tagged WT and mutant TC-GAPDH proteins were purified from *E. coli* DS112 strain (*E. coli* Genetic Resource Center, CT) lacking *gapA* gene. The transformed bacterial cultures were grown in 2 L of M9 minimal medium each to log-phase, containing chloramphenicol and carbenicillin. The cultures were grown at 37 °C to an OD of 0.6 to 0.8 at 37 °C and then were cooled to RT before being induced with 1 mM IPTG. Cultures were shaken at RT and 250 rpm for 48 h before being harvested by centrifugation. Cells were pelleted, then resuspended in lysis buffer (50 mM Tris HCl pH 7.4, 10 mM MgCl_2_, 150 mM NaCl, 10% glycerol, 2 mM BME, protease inhibitor cocktail, DNase I, 1 mg/ml lysozyme) and flash frozen in liquid nitrogen. Lysates were then thawed overnight at 4 °C with rotation before being sonicated and spun in a centrifuge at 4 °C, with the supernatant being collected. Glutathione agarose beads (MCLAB # GAB-300) were equilibrated with wash buffer (50 mM Tris HCl pH 8, 10 mM MgCl_2_, 150 mM NaCl, 5% glycerol, 2 mM BME) and the supernatant was mixed with the beads by gentle shaking for 2 to 3 h at 4 °C (25 ml beads per liter equivalent of cells). The beads were then washed until the flow-through is clear. Thrombin was added to the beads, gently mixed, and then allowed to incubate overnight at 4 °C. After the thrombin incubation, the GAPDH protein was eluted from the beads using cold wash buffer. Benzamidine Sepharose beads were then incubated with the eluted protein for 30 min to remove thrombin. The protein solution was then concentrated in a 10 kDa cut-off centrifugal filter, buffer exchanged if desired, and stored at −80 °C. Purity was assessed by SDS-PAGE, and the protein concentration was determined by Bradford assay (Bio-Rad).

### Heme transfer from purified TC-GAPDH to purified IDO1

TC-GAPDH tetramer (15 μM) was mixed with 30 μM heme at RT for 30 min, and excess heme was removed by passing the solution through a microspin G-25 column (Amersham #27532501). The protein concentration of the recovered protein was measured using the Bradford protein assay (Bio-Rad). Spectra of TC-GAPDH with or without bound heme (5 μM tetramer) and IDO1 (20 μM monomer) were recorded at 250 to 700 nm in a UV-Vis spectrophotometer (Shimadzu). Heme-bound TC-GAPDH (5 μM tetramer) and IDO1 (20 μM monomer) were mixed and incubated at 37 °C for 30 min. After incubation, the protein solution was concentrated using a 10 kDa cut-off centrifugal filter and injected onto an analytical Superdex G-25 gel filtration column attached to an AKTA pure system (Cytiva) for separation. The eluted TC-GAPDH and IDO1 proteins were collected separately based on the elution times of their 280 nm absorbance peaks, and their UV-vis spectra were recorded again. GAPDH-bound ferric heme had absorbance maxima at 415 nm (39), and IDO1-bound ferric heme had absorbance maxima at 404 nm (40). The activity of the IDO1 sample before and after the reaction was measured following a previously published protocol (21). In some cases, the TC-GAPDH was FlAsH-labeled (19) prior to heme binding. Heme loss from FlAsH-TC-GAPDH in the presence or absence of IDO1 was monitored for 30 min at 37 °C in a plate reader (Molecular Devices), with excitation at 508 nm and emission recorded at 528 nm.

### Fluorescence polarization measurements

Alexafluor 488 labeling of Cys groups in purified apo-IDO1 proteins was performed at neutral pH using methods described previously (41). Residual fluorescence polarization measurements were performed at room temperature using methods described previously with modifications (41). Briefly, AF-IDO1 were diluted in 40 mM EPPS/150 mM NaCl/10% glycerol, pH 7.6 buffer to a final concentration of 100 nM in a fluorescent 96 well micro-plate and different amounts of binding partners were added into the wells. The fluorescence polarization of each well was monitored automatically on a Flexstation 3 multimode microplate reader (Molecular Devices) using excitation at 488 nm and emission at 496 nm. All titration experiments were repeated independently three times.

### Protein crosslinking with DSSO

Purified IDO1 protein and rabbit GAPDH protein (Sigma #G2267) in 50 mM HEPES buffer pH 7.4 with 100 mM NaCl was used for the crosslinking reaction. The proteins were allowed to bind to each other at RT for 30 min after which 100-fold excess of the cross linker DSSO (Cayman # 9002863) was added to the proteins and the crosslinking reaction allowed to proceed at RT for 15 min. The reaction was stopped by adding 100 mM Tris-HCl buffer pH 7.4. The crosslinked protein samples were submitted to the Cleveland Clinic proteomics core for MS-based identification of inter- and intra-protein crosslinked residues.

### Identification of intra- and inter-protein crosslinked Lys residues

The lyophilized sample was reconstituted in 100 μl of 6 M urea 0.1 M Tris-HCl pH 8 buffer solution, reduced with DTT and alkylated with iodoacetamide. The solution was diluted with 300 μl of 50 mM ammonium bicarbonate to adjust the pH. The sample was digested with 1.0 μg tryspin at room temperature overnight. The digested samples were desalted by C18 SPE cartridge and dried in a vacuum centrifugal concentrator. It was reconstituted in 30 μl 1% acetic acid for LC-MS analysis. Samples were analyzed by LC-MS using a Fusion Lumos Tribrid MS (Thermo Scientific) equipped with a Dionex Ultimate 3000 nano UHPLC system, and a Dionex (25 cm × 75 μm id) Acclaim Pepmap C_18_, 2-μm, 100-Å reversed-phase capillary chromatography column. Peptide digests (5 μl) were injected and eluted with an acetonitrile/0.1% formic acid gradient at a flow rate of 0.3 μl/min. Experiments were analyzed using two LC-MS/MS methods. The first experiment was a data-dependent analysis to identify the peptides and proteins present in the sample. This experiment involved an initial MS1 scan between 375 to 1700 Da in the orbitrap at a resolution of 120k followed by data dependent MS^2^ scans in the ion trap (3 s cycle time, 35% CE). Dynamic exclusion was enabled where ions within 10 ppm were excluded for 60 s. The second experiment was an MS-Cleavable XL-MS method. The CID-MS^2^-MS^3^ method utilized an initial MS^1^ scan, which measures all ions between 375 to 1500 Da at a resolution of 60k. Peptides identified to have a charge state between 4 and 8 was isolated and subjected to MS^2^ fragmentation using low-energy (25%) collision-induced dissociation (CID) with high-resolution detection in the Orbitrap (30,000 resolution). For DSSO cross-linked peptides, this low-energy MS^2^ fragmentation results in the dissociation of the cross-linked peptides and the resulting ions have a diagnostic mass difference of 31.9721 Da. When this diagnostic mass difference is detected, these fragments are selected for further fragmentation, MS^3^ experiment, in the ion trap. This MS^3^ experiment allows the determination of the peptide sequence including the residues involved in the cross-link. Dynamic exclusion was enabled where ions within 10 ppm were excluded for 60 s. The data dependent data was searched against the sequences of GAPDH (P46406) and IDO (P14902) using Sequest bundled into Proteome Discoverer 2.5 (Thermo Scientific). The parameters used for these searches include oxidation of Methionine and DSSO modification at lysine as variable modifications, protease specificity was full tryptic peptide, tryptic (full) peptides were considered, up to 4 missed cleavages, protein mass tolerance of 10 ppm, and fragment ion mass tolerance of 0.6 Da. Protein and peptide validation was performed using PSM validator. Positively identified proteins require a minimum of 2 peptides, 1 unique, and a 1% false discovery rate (FDR) was applied to identify high confidence proteins. The analysis of the XL-MS data was performed using XLinkX vs 2.2 node bundled into Proteome Discoverer 2.5. The parameters used for these searched include DSSO as the cross-linker, tryptic (full) peptides were considered, semi-proteolytic peptides were considered, precursor mass tolerance of 10 ppm, FTMS fragment ion tolerance of 20 ppm, ITMS Fragment ion tolerance of 0.6 Da, and three missed cleavages. These data were searched against a database generated from the proteins identified in the data-dependent analysis. Validation of the cross-linked peptides was performed using percolator with an FDR set to 0.01. A consensus workflow was also applied for the statistical arrangement. A de-isotope and TopX filter were used to determine the *m*/*z*-error with a selectivity around 10% FDR. All positively identified cross-linked peptides were positively identified.

### IDO1-GAPDH complex modeling

Models of IDO1 complexes were generated using HADDOCK 2.4 (23). A partial crystal structure of Rabbit GAPDH (RCSB ID 1j0x, chains P and R) (18) was used as one of the inputs. We used ColabFold (22) to generate a full-length structural model of human IDO1, guided by the crystal structure of IDO1 with Tryptophan and heme (RCSB ID 6e46) (17). Neither heme nor tryptophan were included in the final IDO1 model used as the other HADDOCK input structure. IDO1 K101 and GAPDH K334 were designated as active residues, and selected surrounding surface residues within a 6 to 10 Å of the active residues were designated as passive. Moreover, we explicitly enforced the experimentally determined (DSSO crosslinked) proximity between K101 and K334 using a restraint table. We visualized and interrogated the HADDOCK output structures using PyMOL v.2 (The PyMOL Molecular Graphics System, Version 2.004, Schrödinger, LLC). Docking of GAPDH and IDO1 structures was also completed using the Chai-1 server (https://lab.chaidiscovery.com) (42). The sequences used for the docking calculations were the human GAPDH tetramer (Uniprot P04406) and human IDO1 monomer (Uniprot P14902). The interaction between GAPDH D141 and IDO1 K101 (<6 Å distance) was included as docking restraint. Calculations were performed both for the basic Chai-1 algorithm and for the Chai-1 algorithm including MSA data. The location of the heme moieties in the GADPH and IDO1 docking structures was calculated superimposing the heme-bound GAPDH model (5) and the IDO1 structure (PDB 4PK5) (43) to the docking models. Figures were produced using PyMOL (44).

### Mutagenesis of the human IDO1 and GAPDH expression plasmids

The single primer site directed mutagenesis protocol (45) was used to design primers which substituted K101 of IDO1 to A and D, E138 in GAPDH was substituted to R, D141 in GAPDH was substituted to R, and both E138 and D141 were substituted to R. Our previously reported WT-IDO1-FLAG mammalian expression plasmid (Sino Biologicals # HG11650-CF) (9) was used as the template for performing the mutagenesis into human IDO1, and our previously reported TC-GAPDH mammalian expression plasmid (19) as the template for the mutagenesis into human GAPDH. All mutated plasmids were confirmed by sequencing and used in transfection of cultured cells in our experiments. The same mutagenesis primers were used to create mutations in bacterial expression plasmids containing His6-IDO1 or GST-TC-GAPDH, and the proteins were expressed and purified from bacterial cultures as described previously (9, 19).

### ^14^C-labeled heme production in cells and measuring radiolabeled heme counts

GlyA-CHO cells were a gift from Dr P. J. Stover, Cornell University, and were confirmed in-house to be glycine-auxotrophic for growth. The protocol followed for administering ^14^C-δ-ALA to cells, the IP pull-down of IDO1, and radiolabel counting has been described in (5, 21).

### Transfection and expression of human proteins in mammalian cells

Human IDO1-FLAG (Sino Biologicals # HG11650-CF) and variant plasmids, human TC-GAPDH and variant plasmids, and human TDO-FLAG (Sino Biologicals # HG13215-CF) were transfected in HEK293T cells (ATCC # CRL-11268) using Lipofectamine2000 (Invitrogen # 11668019). Briefly, cells were grown in phenol red free DMEM medium containing 10% of either normal serum or heme-depleted fetal bovine serum. In some cases, Succinyl acetone (SA; Sigma # D-1415) which inhibits endogenous heme biosynthesis was used at 400 μM to treat cells for 72 h in DMEM containing heme-depleted serum. Cells were seeded into appropriate plates, and the next day were transfected with desired plasmids. This involved transfecting cells with the IDO1-FLAG expression plasmids (either WT or mutants) alone or in combination with the HA-TC-GAPDH plasmids (either WT or mutants). The protein expression was allowed to continue for 36 h, in some cases in the presence of SA, after which cycloheximide (Chx, Sigma #C7698) was used to treat cells at 5 μg/ml for 12 h to inhibit further protein synthesis. Cells were then utilized for further experiments. In some cases, mitochondrial heme synthesis was restarted by removing SA and adding in 1 mM δ -ALA and 10 μM Ferric citrate and its binding to FlAsH-labeled TC-GAPDH in live cells were monitored as described in (19).

### Transfection of siRNA and gene silencing

Cell GAPDH protein expression was reduced using siRNA against human GAPDH mRNA. Commercially available siRNA against human GAPDH (# D-001830-01-05) and scrambled siRNA (# D-001810-10-05) were purchased from Horizon Discovery Biosciences and used at a final concentration of 50 nM in cultures of mammalian cells of low passage number along with Lipofectamine2000. The siRNA-treated cells were cultured for 48 h before they received transfections with protein expression plasmids as described earlier.

### IDO1 and TDO activity assay from cell culture medium

The enzyme activity of IDO1 and TDO was measured using a colorimetric assay (9). The cells after treatment with Chx were treated with substrate L-Trp at 2 mM in phenol red free DMEM medium containing appropriate type of serum (normal or heme depleted) for 6 h to allow substrate utilization and L-Kynurenine product formation. The medium was collected and de-proteinized by adding an equal volume of 30% trichloroacetic acid (TCA) and incubated at 50 °C for 30 min. The tubes were centrifuged at 9000 g for 10 min at room temperature to precipitate proteins from the medium. Equal volumes of de-proteinized sample were mixed with a 20 mg/ml solution of p-dimethyl-amino-benzaldehyde (Ehrlich’s reagent; Sigma # 109762) in glacial acetic acid at room temperature to allow the formation of a yellow-colored product. The end point absorbance of this product was measured at 492 nm (Molecular Devices) to determine the concentration of L-Kyn in the medium. A standard curve was obtained using commercial L-Kyn (Sigma #K8625) dissolved in 0.5 N hydrochloric acid in various concentrations using the exact same method.

### IDO1 and TDO activity assay in cell supernatants

The activity of IDO1 and TDO in cell supernatants were measured as described in (46) with modifications. Briefly, 500 μg of cell supernatant protein was added to a 1 ml reaction containing 50 mM potassium phosphate buffer (pH 6.5), 20 mM ascorbate, 10 μM methylene blue, 100 μg/ml catalase, and 1 mM L-Trp and incubated at 37 ° C for 30 min. The reactions were stopped by adding 1 ml of 30% TCA to denature the proteins. The next steps were followed as per the previously described colorimetric detection method of Kyn using Ehrlich’s reagent. In some cases, cell supernatants with IDO1 were given heme prior to assay. Supernatants (1 mg protein) were made in 1, 3, or 6 μM heme, incubated at room temperature for 1 h, passed through a desalting column (Sephadex G-25 resin), and then assayed for activity as described earlier.

### Immunoprecipitation and Western blot

Cells were lysed using 50 mM Tris-HCl pH 7.4 buffer with 0.1% Triton X-100, 100 mM NaCl and EDTA-free protease inhibitor cocktail (Roche). Protein concentration was measured using the Bradford method (Bio-Rad # 500–0006). IP pull-downs were performed using 1 mg of whole cell extracts with anti-FLAG antibody (Sigma #F1804). Protein G agarose beads (Millipore # 16–201) were used to pull down the antibody-protein complex. The beads were washed well with the lysis buffer. For ^14^C heme count experiment, 5 ml of scintillation fluid was added to the beads and heme counts were measured on a scintillation counter. For other experiments, proteins on beads were boiled in Laemmli buffer, resolved onto 10% or 15% SDS-PAGE and transferred to PVDF membrane (Bio-Rad # 1620177) and probed for proteins of interest. Western blot was performed with anti-FLAG (Sigma #F1804; dilution 1:1000), anti-IDO (Cell Signaling # 86630S; dilution 1:1000), anti-GAPDH (Santa Cruz Biotechnology # sc-32233; dilution 1:2500) and anti-α-Tubulin (Santa Cruz Biotechnology # sc-5286; dilution 1:2500). The proteins were detected using chemiluminescence using HRP-conjugated secondary antibodies of either anti-mouse (Bio-Rad # 170–6516, dilution 1:10,000) or anti-rabbit (GE Healthcare # NA9340, dilution 1:10,000) origin and ECL substrate (Thermo Scientific # 32106). The images were acquired using a chemidoc system from Bio-Rad. Densitometry analyses on the images were performed using ImageJ software (47).

### Heme titration of TC-GAPDH in cell supernatants

HEK293T cells were grown in DMEM containing 10% serum. Cells were allowed to express the HA-TC-GAPDH (WT or mutants) for 48 h post transfection of plasmid. Cells were then incubated with 1 μM FlAsH dye (Cayman # 20704) in Opti-MEM medium for 30 min at RT protected from light. Afterward, cells were washed three times with phenol red-free DMEM. Cells were lysed using ice cold 50 mM MOPS, 150 mM NaCl, pH 7.4 and EDTA-free protease inhibitor cocktail (Roche) and lysates spun at 4 °C at 10,000×*g* for 10 min to yield supernatants for analysis. Protein concentration was measured using the Bradford method (Bio-Rad # 500–0006). 0.08 mg/ml of supernatant protein was mixed in buffer (50 mM MOPS, 150 mM NaCl, pH 7.4) at RT with varying concentrations of heme (0–2 μM), which were prepared from a stock solution of hemin chloride in DMSO. After mixing the reaction was allowed to equilibrate for 30 min in the dark. The end point fluorescence signals were measured in the 96-well plate reader at RT using excitation at 508 nm and emission at 528 nm.

### Multiple sequence alignment of GAPDH and IDO1 protein sequences

We obtained GAPDH protein sequences from different species (5) from the Uniprot database and performed multiple sequence alignment using Clustal Omega (48). Briefly, the GAPDH sequences used were from *E. coli* K12 *gapA*, *Trypanosoma brucei* GAPDH, *Drosophila melanogaster* GAPDH1, *Danio rerio* GAPDH, *Homo sapiens* GAPDH, *Oryctolagus cuniculus* GAPDH, *Saccharomyces cerevisiae* GAPDH *TDH3* and *Arabidopsis thaliana* GAPDH *GAPC1*. Only three species out of all mentioned above express IDO1, namely *D. rerio, H. sapiens* and *O. cuniculus.* The IDO1 protein sequences were obtained similarly from the Uniprot database and performed multiple sequence alignment using Clustal Omega (48). Secondary structures were aligned with the multiple sequence alignments of both GAPDH and IDO1 using ESPript3.0 (49).

### Statistical analysis

All experiments were done in three independent trials, with three replicates per trial. The results are presented as the mean of the three trial values ± standard deviation. The statistical test used to measure significance (*p*-values) was Student's *t* test in the software GraphPad Prism (v9).

## Data availability

All data are contained within this manuscript or are available from the authors.

## Supporting information

This article contains supporting information.

## Conflict of interest

The authors declare that they have no conflicts of interest with the contents of this article.
