# Supplementary material for: GAPDH heme delivery to Indoleamine 2,3-dioxygenase 1 involves their complex formation and complementary charge pairing at the protein-protein interface
Source: J Biol Chem. 2025 Jul 1;301(8):110443. doi: 10.1016/j.jbc.2025.110443 (PMC12329532; doi:10.1016/j.jbc.2025.110443)
Supplement: Supplemental data [file mmc1.pdf]

GAPDH heme delivery to Indoleamine 2,3-dioxygenase 1 involves their complex formation and complementary charge pairing at the protein-protein interface

Pranjal Biswas[1], Yue Dai[1], Dhanya T. Jayaram[1], Priya Das Sinha[1], Saurav Misra[2], Jesus Tejero[3,4], Belinda Willard[5], and Dennis J. Stuehr[1]*

Number	Page number
Fig. S1	S1
Fig. S2	S2
Table S1	S3
Table S2	S4-S6
Table S3	S7
Fig. S3	S8
Fig. S4	S9
Fig. S5	S10
Fig. S6	S11
Fig. S7	S12
Fig. S8	S13
Fig. S9	S14
Fig. S10	S15
Fig. S11	S16
Fig. S12	S17
Fig. S13	S18
Fig. S14	S19
Fig. S15	S20
Fig. S16	S21

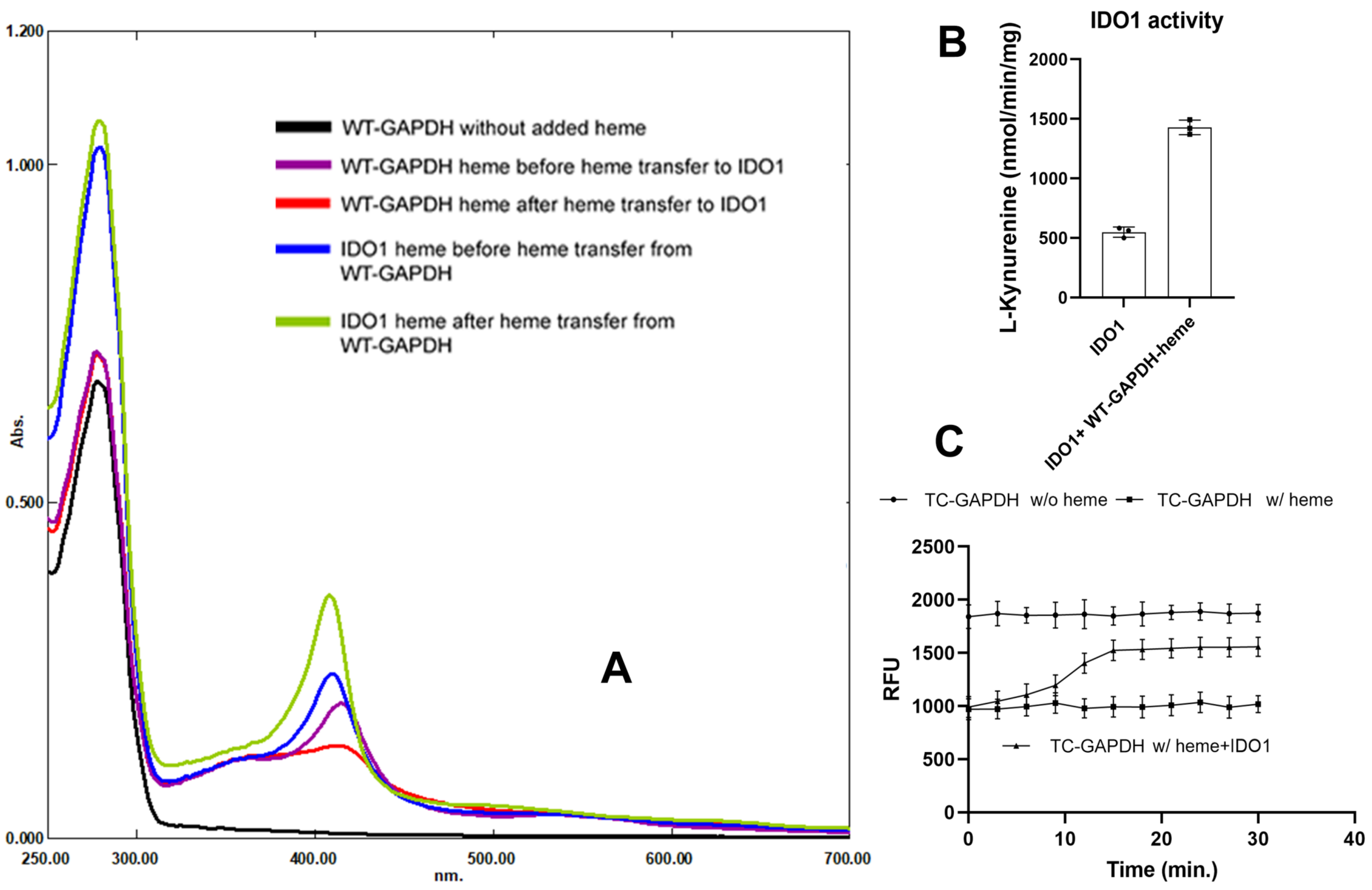

Fig. S1: *In vitro* heme transfer from purified WT-TC-GAPDH to purified His-IDO1. (A) Spectra of WT-TC-GAPDH heme free, heme bound, and after heme transfer to IDO1. Spectra of IDO1 before and after heme transfer from WT-TC-GAPDH. (B) Activity of IDO1 before and after heme transfer from WT-TC-GAPDH. (C) Change in the fluorescence emission intensity of FlAsH-labeled WT-TC-GAPDH versus time during incubations at 37 °C for its heme free or heme bound versions incubated alone, and for its heme bound version incubated with apo-IDO1. Data is shown as mean ± s.e.m., n=3.

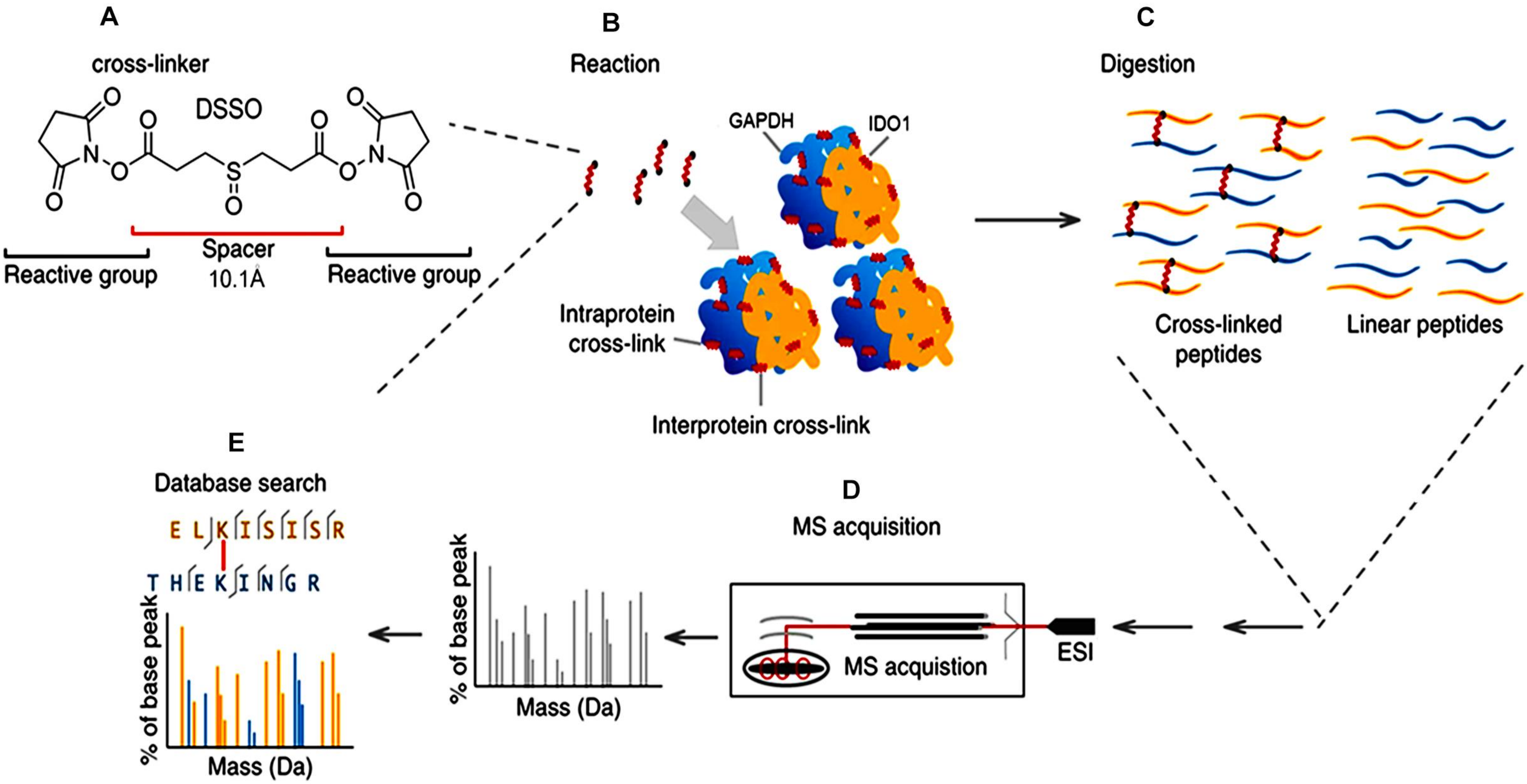

Fig. S2: Workflow of protein crosslinking with DSSO and downstream identification of crosslinked Lys residues by mass spectroscopy. (A) Structure of DSSO crosslinker showing NHS ester reactive groups and spacer having length of 10.1 Å. (B) Cartoon showing interaction complex between IDO1 and GAPDH crosslinked by DSSO. (C) Crosslinked protein complexes were digested with proteases resulting in both crosslinked peptides and un-crosslinked peptides from both proteins. (D) Mass spectroscopic data from the peptides were obtained. (E) Database searches were performed based on the masses of Lys crosslinked residues to identify the position of the crosslinked Lys and sequence of the crosslinked peptides.

Table S1 Summary of results

Sample Name	Sample number	Protein	Accession	Gene ID	Mass Da	Pep #	Pep Uni	Seq. Cov %	Sequest Score
3.1.21 GAPDH IDO1_1	SB19-89-37	Glyceraldehyde-3-phosphate dehydrogenase	P46406	Gapdh	36	51	51	84%	1080.3
		Indoleamine 2,3-dioxygenase 1	P14902	IDO1	45	51	51	84%	777.9
3.1.21 GAPDH IDO1_2	SB19-89-38	Glyceraldehyde-3-phosphate dehydrogenase	P46406	Gapdh	36	42	42	82%	852.1
		Indoleamine 2,3-dioxygenase 1	P14902	IDO1	45	56	56	85%	588.8

Table S2. Crosslinked peptides identified in GAPDH-IDO1 samples.

[M+H]	m/z	z	Δm ppm	Ret Time Min	XL type	Site A	Site B	Protein A	Protein B	Sequence A	Sequence B	XlinkX score GAPDH IDO1_1	XlinkX score GAPDH IDO1_2
2128.096	532.7794	4	57.1979	0.22032	Inter	332	101	GAPDH	IDO1	VVDLMVHMASK*E	K*VLPR	47.63	ni
2494.382	624.351	4	49.8936	-0.48968	Inter	213	101	GAPDH	IDO1	GAAQNIIPASTGAAK*AVGK	K*VLPR	86.8	ni
2708.325	677.8368	4	61.4401	0.19712	Inter	332	397	GAPDH	IDO1	VVDLMVHMASK*E	STTEK*SLLKEG	181.44	ni
1677.886	420.227	4	35.5887	0.34371	Intra	2	115	GAPDH	GAPDH	VK*VGVNGFGR	GGAK*R	166.07	123.75
1787.943	447.7412	4	29.7283	0.86742	Intra	64	115	GAPDH	GAPDH	AENGK*LVINGK	GGAK*R	147.28	ni
1847.935	462.7393	4	26.8725	-0.24619	Intra	ni	ni	GAPDH	GAPDH	AGAHLK*GGAK	YDDIK*K	ni	145.72
1870.021	468.2607	4	41.2899	0.36373	Intra	217	115	GAPDH	GAPDH	AVGK*VIPELNGK	GGAK*R	143.5	ni
1971.006	493.5068	4	45.431	0.188	Intra	2	257	GAPDH	GAPDH	VK*VGVNGFGR	YDDIK*K	71.22	ni
1989.984	498.2515	4	33.7034	-0.84962	Intra	252	257	GAPDH	GAPDH	AAK*YDDIKK	YDDIK*K	167.23	105.1
2094.087	524.2772	4	37.6322	-1.76136	Intra	261	257	GAPDH	GAPDH	VVK*QASEGPLK	YDDIK*K	181.87	155
2099.118	525.5349	4	38.5628	0.2714	Intra	2	111	GAPDH	GAPDH	VK*VGVNGFGR	AGAHLK*GGAK	123.86	109.44
2118.104	530.2814	4	27.8341	-0.20042	Intra	252	111	GAPDH	GAPDH	AAK*YDDIKK	AGAHLK*GGAK	130.75	ni
2154.056	539.2694	4	48.4367	-1.3661	Intra	192	257	GAPDH	GAPDH	TVDGPSGK*LWR	YDDIK*K	155.11	ni
2174.164	544.2964	4	33.602	-0.62479	Intra	ni	ni	GAPDH	GAPDH	YDDIKK*VVK	AGAHLK*GGAK	ni	192.32
2222.194	556.3039	4	31.4458	-0.01101	Intra	261	111	GAPDH	GAPDH	VVK*QASEGPLK	AGAHLK*GGAK	190.57	ni
2254.152	564.2936	4	25.1074	0.77128	Intra	59	111	GAPDH	GAPDH	FHGTVK*AENGK	AGAHLK*GGAK	145.72	ni
2255.221	451.8501	5	35.7615	-1.06739	Intra	111	2	GAPDH	GAPDH	AGAHLK*GGAKR	VK*VGVNGFGR	ni	135.05
2297.089	575.0276	4	55.9456	0.34624	Intra	332	257	GAPDH	GAPDH	VVDLMVHMASK*E	YDDIK*K	123.03	ni
2332.243	583.8162	4	51.1054	-0.12497	Intra	64	2	GAPDH	GAPDH	AENGK*LVINGK	VK*VGVNGFGR	101.71	ni
2364.25	591.8179	4	36.5035	-0.484	Intra	261	252	GAPDH	GAPDH	VVK*QASEGPLK	AAK*YDDIKK	211.53	ni
2441.198	611.0551	4	43.4928	0.89396	Intra	332	111	GAPDH	GAPDH	VVDLMVHMAS*KE	AGAHLK*GGAK	145.09	ni

2515.301	629.5808	4	53.5626	-1.73736	Intra	192	64	GAPDH	GAPDH	TVDGPSGK*LWR	AENGK*LVINGK	166.58	ni
2581.303	646.0811	4	44.9262	0.06131	Intra	332	115	GAPDH	GAPDH	VVDLMVHMASK*E	AGAHLKGGAK*R	160.89	ni
2658.34	665.3404	4	60.59	-1.11769	Intra	332	64	GAPDH	GAPDH	VVDLMVHMASK*E	AENGK*LVINGK	159.43	ni
2671.352	668.5935	4	57.1059	-0.45254	Intra	332	261	GAPDH	GAPDH	VVDLMVHMASK*E	VVK*QASEGPLK	188.49	ni
2747.329	687.5876	4	60.993	-2.3319	Intra	332	192	GAPDH	GAPDH	VVDLMVHMASK*E	TVDGPSGK*LWR	235.01	ni
2791.491	698.6282	4	42.8541	1.04141	Intra	213	111	GAPDH	GAPDH	GAAQNIIPASTGAAK*AVGK	AGAHLK*GGAK	177.36	ni
2914.556	729.3945	4	56.3953	-0.13545	Intra	213	2	GAPDH	GAPDH	GAAQNIIPASTGAAK*AVGK	VK*VGVNGFGR	144.65	ni
2916.443	729.8663	4	56.0124	0.18185	Intra	192	192	GAPDH	GAPDH	TVDGPSGK*LWRDGR	TVDGPSGK*LWR	136.02	ni
2925.526	732.137	4	56.9902	-0.27664	Intra	192	217	GAPDH	GAPDH	TVDGPSGK*LWRDGR	AVGK*VIPELNGK	148.9	ni
2933.543	734.1411	4	46.7367	0.23637	Intra	213	252	GAPDH	GAPDH	GAAQNIIPASTGAAK*AVGK	AAK*YDDIKK	220.7	ni
3024.612	756.9085	4	53.0866	-0.03959	Intra	213	64	GAPDH	GAPDH	GAAQNIIPASTGAAK*AVGK	AENGK*LVINGK	149.57	ni
3059.475	765.6243	4	61.98	-0.32956	Intra	192	332	GAPDH	GAPDH	TVDGPSGK*LWRDGR	VVDLMVHMASK*E	210.98	ni
3097.608	775.1575	4	58.4549	-0.43977	Intra	213	192	GAPDH	GAPDH	GAAQNIIPASTGAAK*AVGK	TVDGPSGK*LWR	151.35	196.06
3240.654	810.9189	4	63.7465	0.04276	Intra	213	332	GAPDH	GAPDH	GAAQNIIPASTGAAK*AVGK	VVDLMVHMASK*E	199.21	ni
3425.759	857.1953	4	55.1322	-0.99058	Intra	213	192	GAPDH	GAPDH	GAAQNIIPASTGAAK*AVGK	TVDGPSGK*LWRDGR	289.85	211.51
3429.872	858.2235	4	69.4864	0.40245	Intra	70	2	GAPDH	GAPDH	LVINGK*AITIFQERDPANIK	VK*VGVNGFGR	206.73	198.67
3461.842	693.1742	5	66.3134	-0.8811	Intra	249	258	GAPDH	GAPDH	VPTPNVSVVDLTCRLEK*AAK	YDDIKK*VVK	244.63	244.68
3606.771	902.4482	4	55.9928	-0.36656	Intra	143	111	GAPDH	GAPDH	YDNSLK*IVSNASCTTNCLAPLAK	AGAHLK*GGAK	149.72	169.44
3935.063	984.5212	4	56.4548	-0.52314	Intra	213	213	GAPDH	GAPDH	DGRGAAQNIIPASTGAAK*AVGK	GAAQNIIPASTGAAK*AVGK	190.57	ni
4156.072	832.0202	5	59.9699	0.71154	Intra	137	111	GAPDH	GAPDH	RVIISAPSADAPMFVMGVNHEK*YDNSLK	AGAHLK*GGAK	162.81	ni
4449.138	890.6335	5	76.7911	-2.1406	Intra	137	332	GAPDH	GAPDH	VIISAPSADAPMFVMGVNHEK*YDNSLK	VVDLMVHMASK*E	171.32	ni
4741.495	949.1047	5	83.2122	-1.01393	Intra	184	217	GAPDH	GAPDH	VIHDHFGIVEGLMTTVHAITATQK*TVDGPSGK	AVGK*VIPELNGK	ni	241.41
4875.463	975.8984	5	86.6858	-0.298	Intra	184	332	GAPDH	GAPDH	VIHDHFGIVEGLMTTVHAITATQK*TVDGPSGK	VVDLMVHMASK*E	190.92	ni

2641.341	661.0906	4	59.5005	-0.97449	Intra	323	397	IDO1	IDO1	EFVLSK*GDAGLR	STTEK*SLLKEG	101.35	ni
2997.517	600.3092	5	60.5045	-2.64529	Intra	323	101	IDO1	IDO1	EFVLSK*GDAGLREAYDACVK	K*VLPR	112.68	ni
3709.881	742.782	5	70.3058	-0.36525	Intra	94	397	IDO1	IDO1	LVLGCITMAYVWGK*GHGDVRK	STTEK*SLLKEG	119.03	ni
2464.29	616.8279	4	65.7921	1.172	Intra	377	101	IDO1	IDO1	LEAK*GTGGTDLMNFLK	K*VLPR	161.04	ni
3342.625	669.3307	5	66.2488	-2.70665	Intra	389	366	IDO1	IDO1	GTGGTDLMNFLK*TVR	ENK*TSEDPSKLEAK	188.12	ni
2959.474	740.6239	4	73.9438	0.38501	Intra	389	397	IDO1	IDO1	GTGGTDLMNFLK*TVR	STTEK*SLLKEG	244.68	297.54
2971.437	743.6147	4	71.0464	-1.47426	Intra	389	373	IDO1	IDO1	GTGGTDLMNFLK*TVR	TSEDPSK*LEAK	251.64	167.61
2345.202	587.0559	4	36.8352	0.53118	Intra	366	101	IDO1	IDO1	ENK*TSEDPSKLEAK	K*VLPR	309.78	ni
2773.411	694.1081	4	73.5864	-0.36208	Intra	389	397	IDO1	IDO1	GTGGTDLMNFLK*TVR	STTEK*SLLK	329.46	260.25
3044.506	761.8821	4	69.1242	0.18062	Intra	377	397	IDO1	IDO1	LEAK*GTGGTDLMNFLK	STTEK*SLLKEG	ni	175.6
3400.732	850.9385	4	70.2923	-0.21364	Intra	389	397	IDO1	IDO1	LEAKGTGGTDLMNFL*KTVR	STTEK*SLLKEG	ni	230.19

Table S3: Number of different peptides identified after crosslinking

	GAPDH-IDO1_1	GAPDH-IDO1_2
Intrapeptide GADPH	37	13
Intrapeptide IDO1	9	5
Interpeptide GAPDH-IDO1	3	0

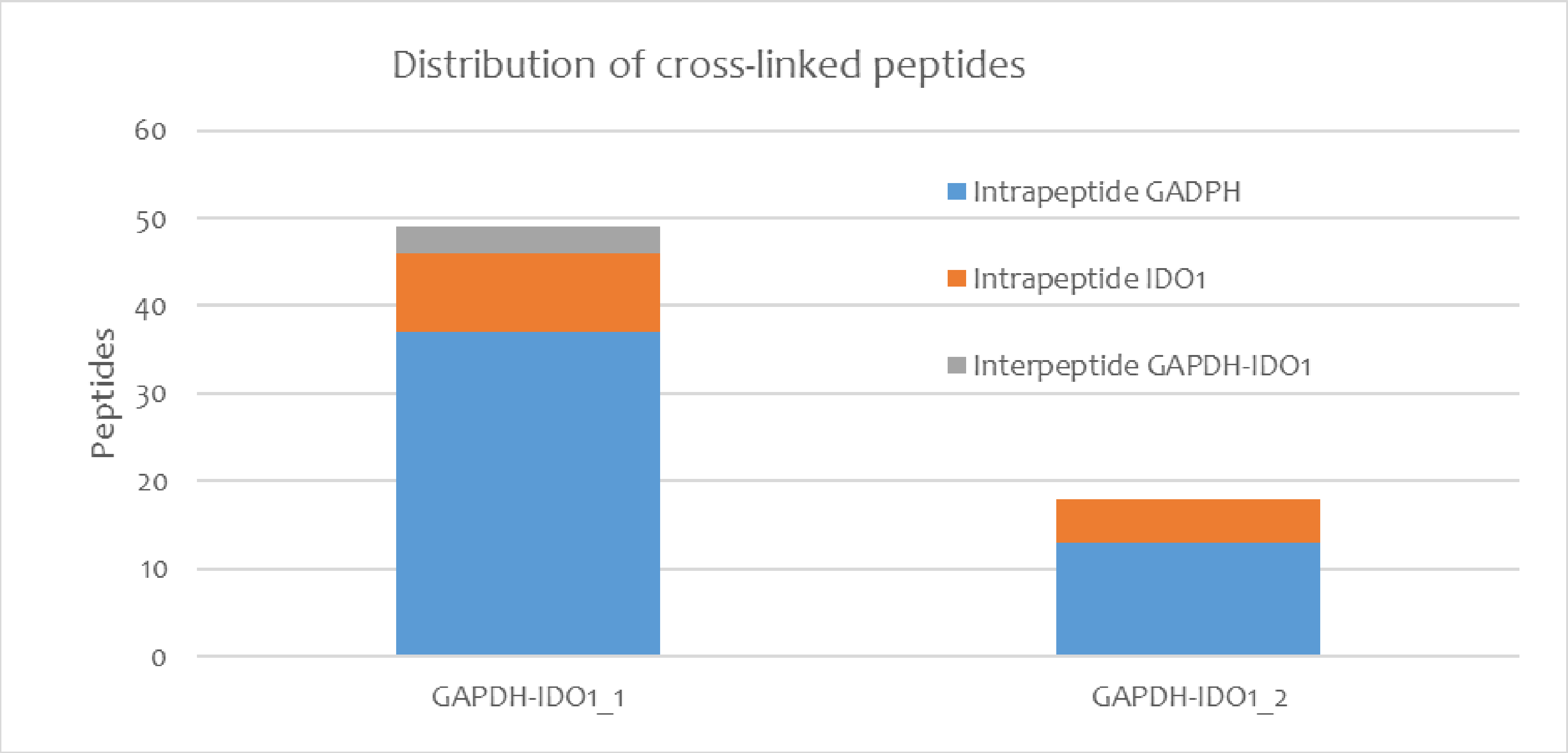

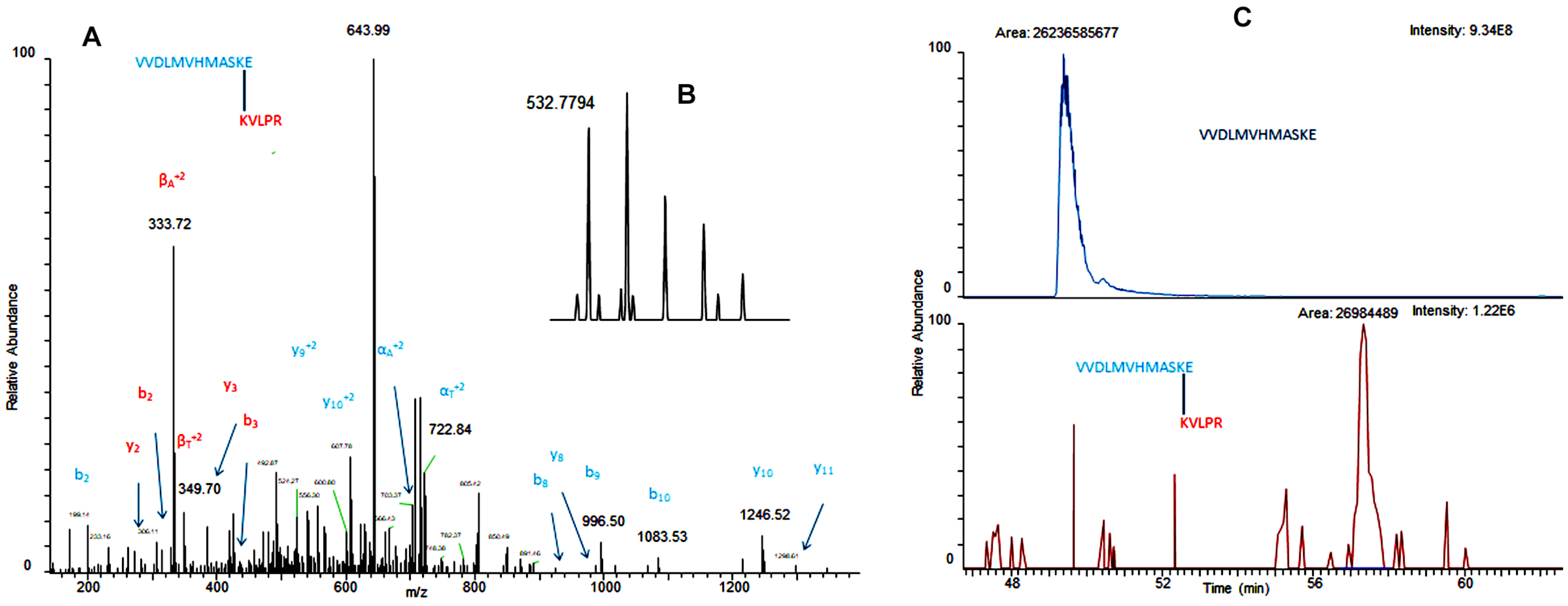

Fig. S3: (A) The MS/MS spectra for the 533.7794 Da ions is consistent with the GAPDH_K332-IDO_K101 cross-linked peptide and contains both the thiol (αT, βY) and alkene (αA, βA) forms as well as several sequence specific b and y ions from each peptide. A quadruply charged peptide with an observed m/z of 532.7794 Da (insert B) was identified and has a mass within 0.22 ppm of the expected mass for the cross-linked peptide between the GAPDH derived peptide (322)VVDLMVHMASK*E(333) and the IDO1 derived peptide (101)K*VLPR(105). (C) Chromatograms for the unmodified GAPDH derived peptide, VVDLMVHMASKE, and the cross-linked peptides are shown.

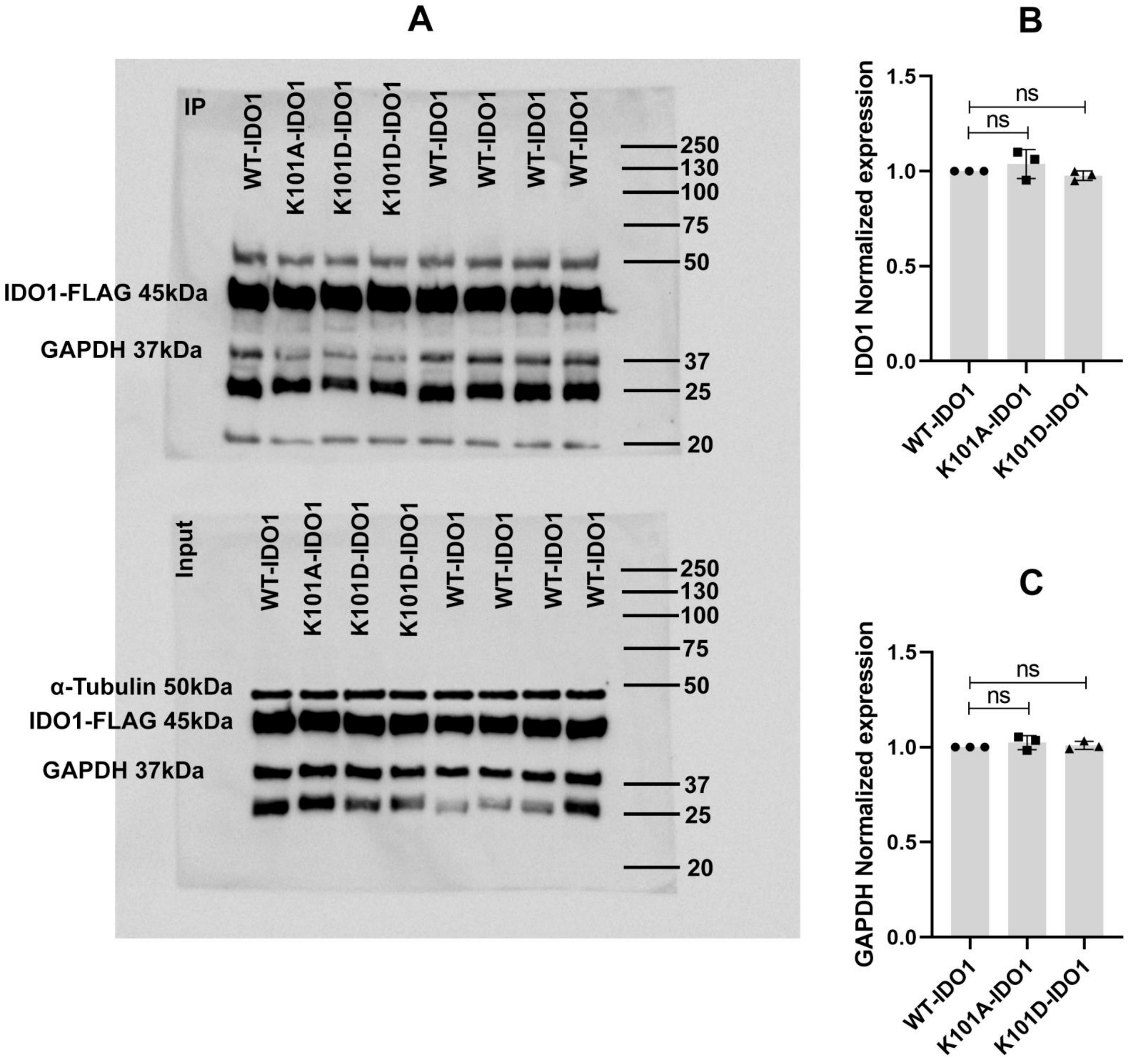

Fig. S4: Interaction of WT-, K101A- and K101D-IDO1 with endogenous GAPDH in HEK293T cells. (A) Full IP blot showing levels of GAPDH co-IP'd with IP'd IDO1-FLAG. Full input blot shows the protein expression levels of the different IDO1 proteins and GAPDH. The cropped image of the IP and input blot has been shown in Fig.1A. (B) IDO1 expression was normalized to that of α-Tubulin using densitometry. (C) GAPDH expression was normalized to that of α-Tubulin using densitometry. Data is shown as mean ± s.d., n=3. ns, not significant.

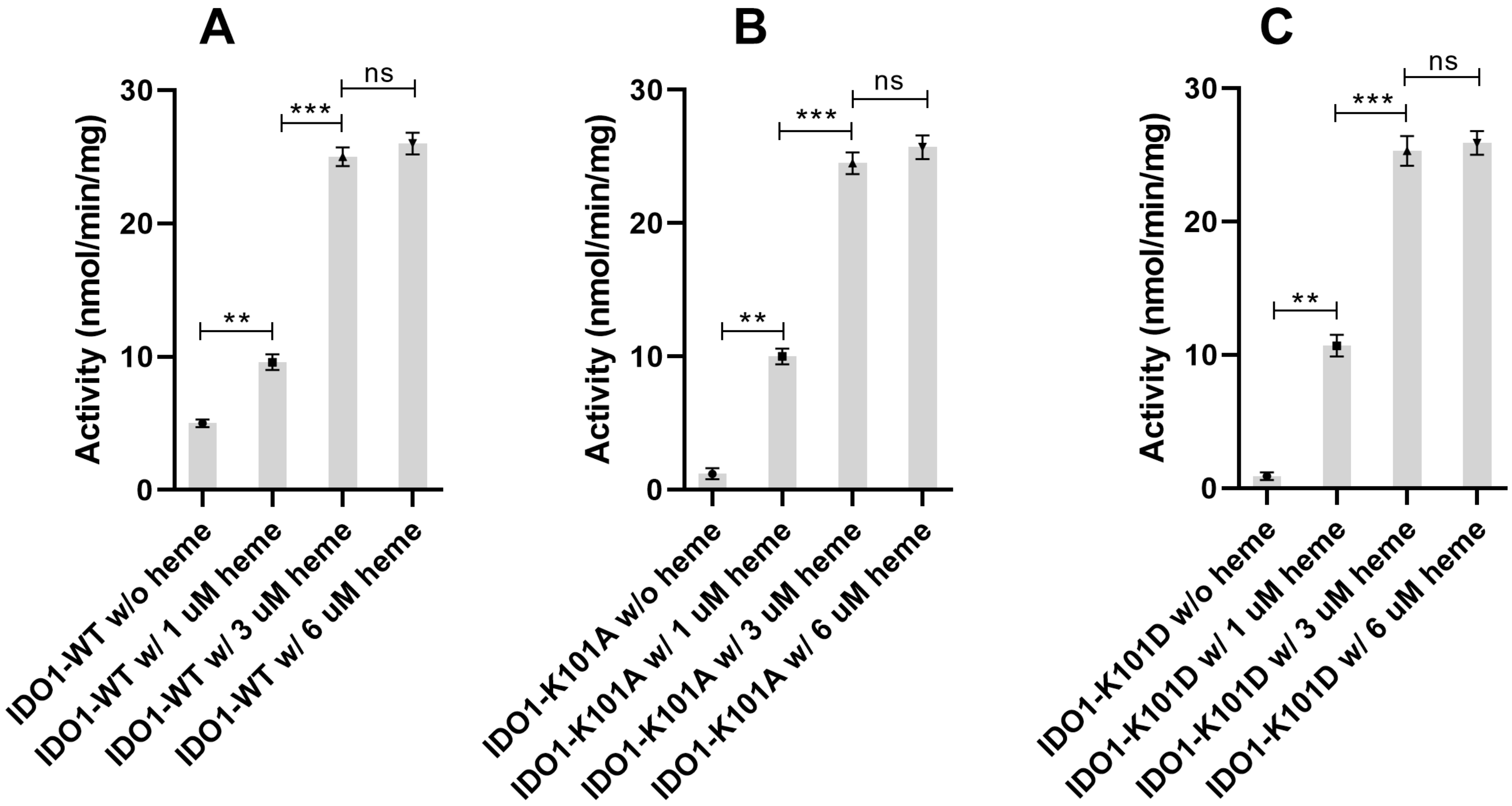

Fig. S5: Heme titrations of WT-, K101A- and K101D-IDO1 in HEK293T cell supernatants. (A) WT-IDO1 expressing cell supernatants were titrated with 0, 1, 3 and 6 μM of hemin chloride at RT and the resulting IDO1 activity was measured. (B) Similar assay with K101A-IDO1 expressing cell supernatants. (C) Similar assay with K101D-IDO1 expressing cell supernatants. Data is shown as mean ± s.d., n=3. $^{**}p<0.01$, $^{***}p<0.001$, ns, not significant.

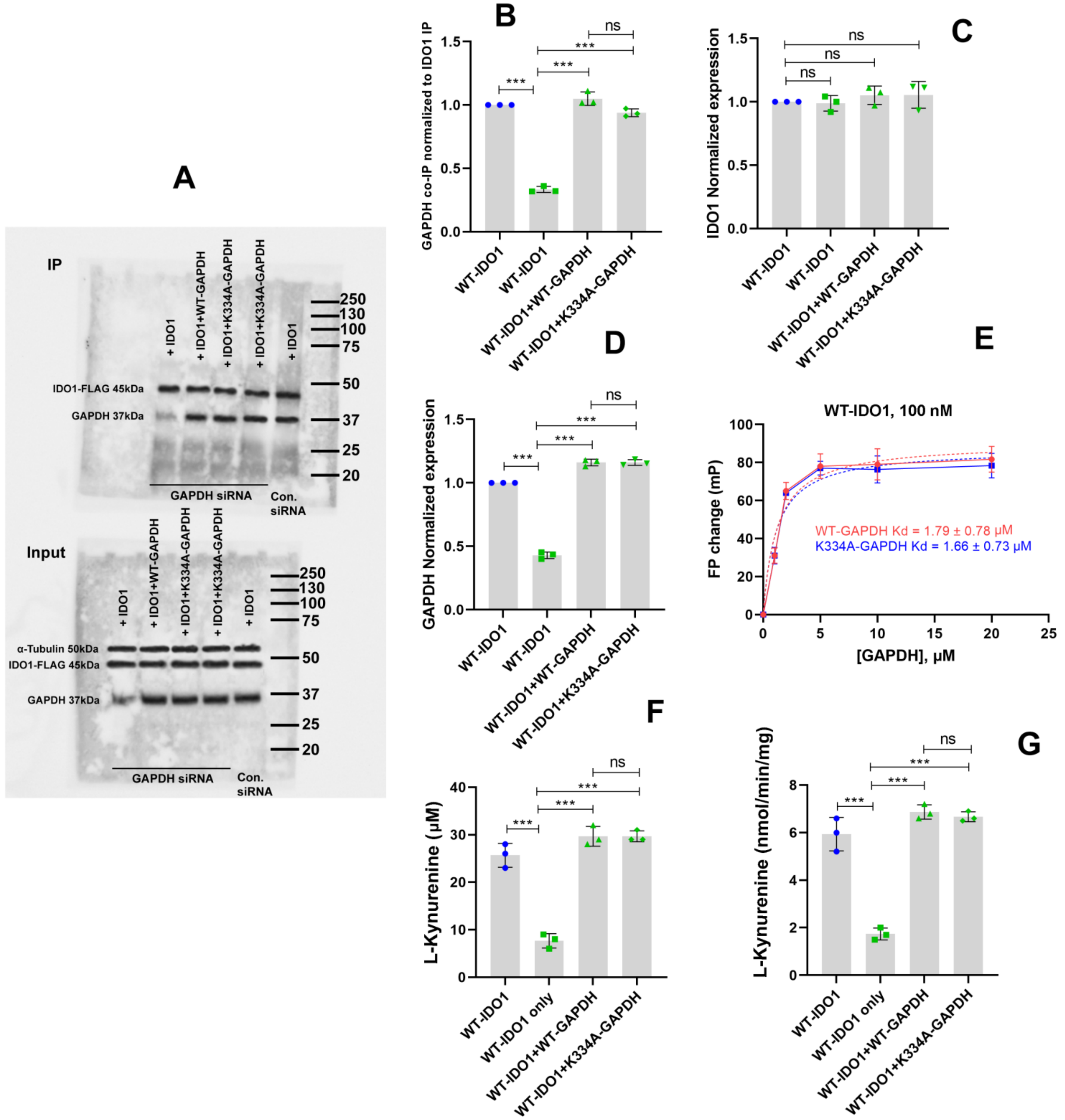

Fig. S6: Interaction of WT-IDO1 with WT-GAPDH and K334A-GAPDH in HEK293T cells treated with control siRNA (blue) and GAPDH siRNA (green). (A) IP blot showing levels of different GAPDH co-IP'd with IP'd IDO1-FLAG. Input blot shows the protein expression levels of the IDO1 proteins and the silencing of endogenous GAPDH and expression of different GAPDH from siRNA resistant plasmids. (B) GAPDH co-IP'd with IP'd IDO1-FLAG was normalized to that IP'd IDO1 using densitometry. (C) IDO1 expression was normalized to that of α-Tubulin using densitometry. (D) GAPDH expression was normalized to that of α-Tubulin using densitometry. (E) Interaction between purified WT-IDO1 and WT/K334A-GAPDH was measured using fluorescence polarization. (F) Activity of WT-IDO1 in medium of HEK293T cells treated with control siRNA (blue) and GAPDH siRNA (green) and rescued with WT/K334A-GAPDH. (G) Activity of WT-IDO1 in supernatant of HEK293T cells under the same experimental conditions. Data is shown as mean ± s.d., n=3. ****p*<0.001, ns, not significant.

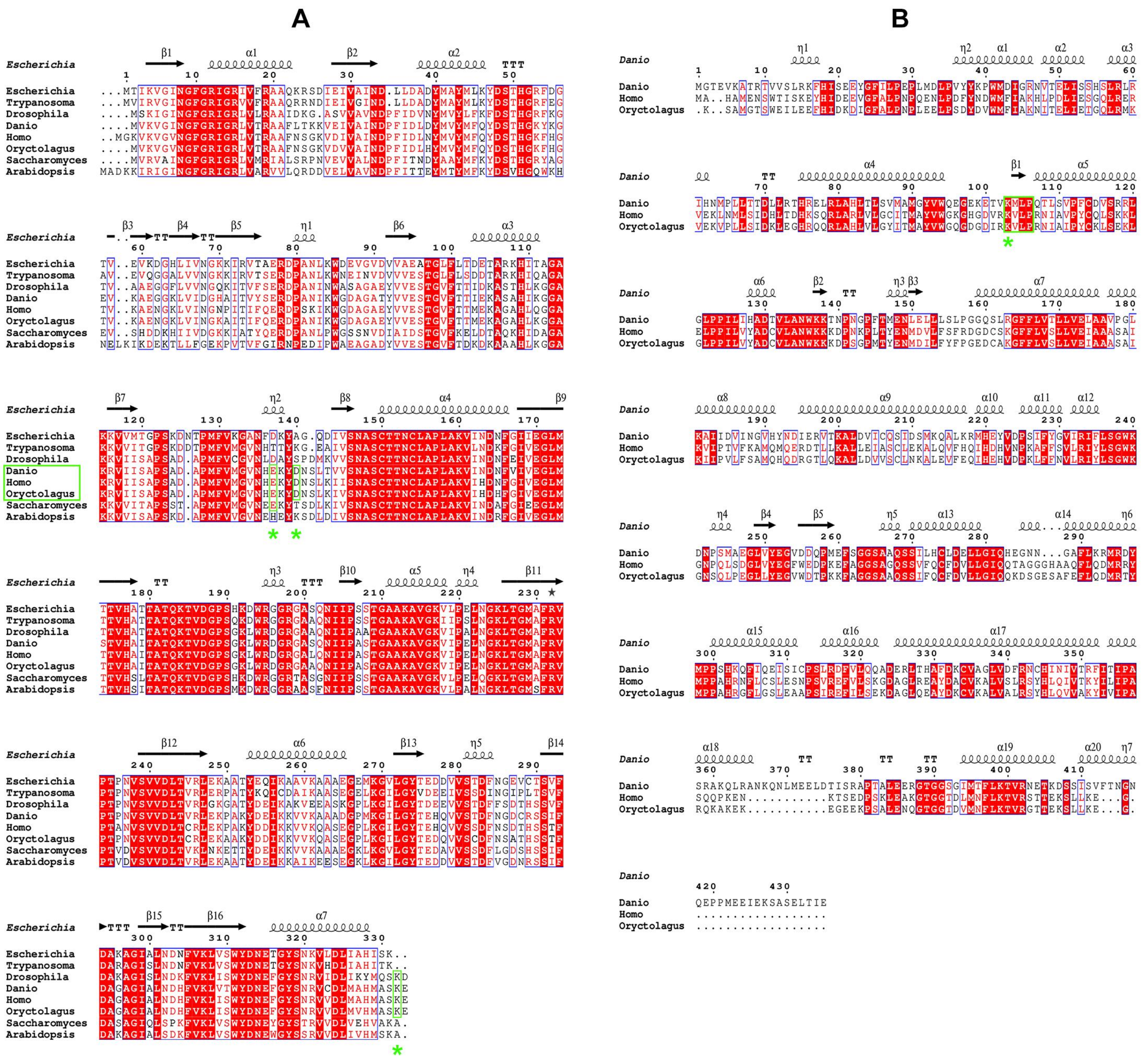

Fig. S7: Multiple sequence alignment of (A) GAPDH protein from different species showing that E138, D141 and K334 (marked in green) is conserved in *Danio rerio*, *Homo sapiens* and *Oryctolagus cuniculus*. (B) Out of all the species considered only *Danio rerio*, *Homo sapiens* and *Oryctolagus cuniculus* express IDO1 in which K101 is highly conserved (marked in green).

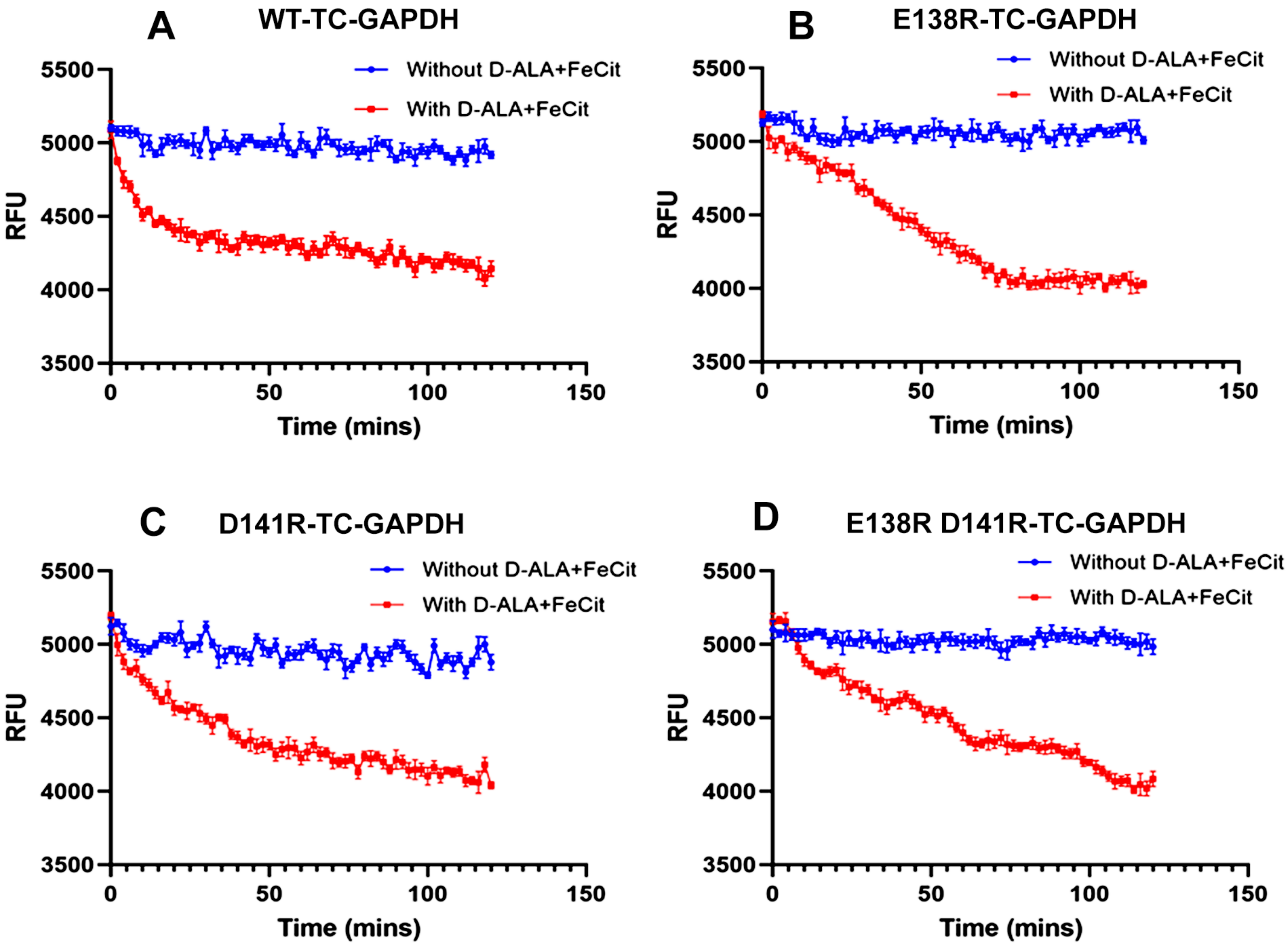

Fig. S8: Binding of mitochondria generated heme by WT- and various mutants of FlAsH-bound TC-GAPDH expressed in HEK293T cells as evidenced by quenching of the FlAsH fluorescence upon TC-GAPDH heme binding. (A) WT-TC-GAPDH. (B) E138R-TC-GAPDH. (C) D141R-TC-GAPDH. (D) E138R D141R-TC-GAPDH. Data is shown as mean ± s.d., n=3.

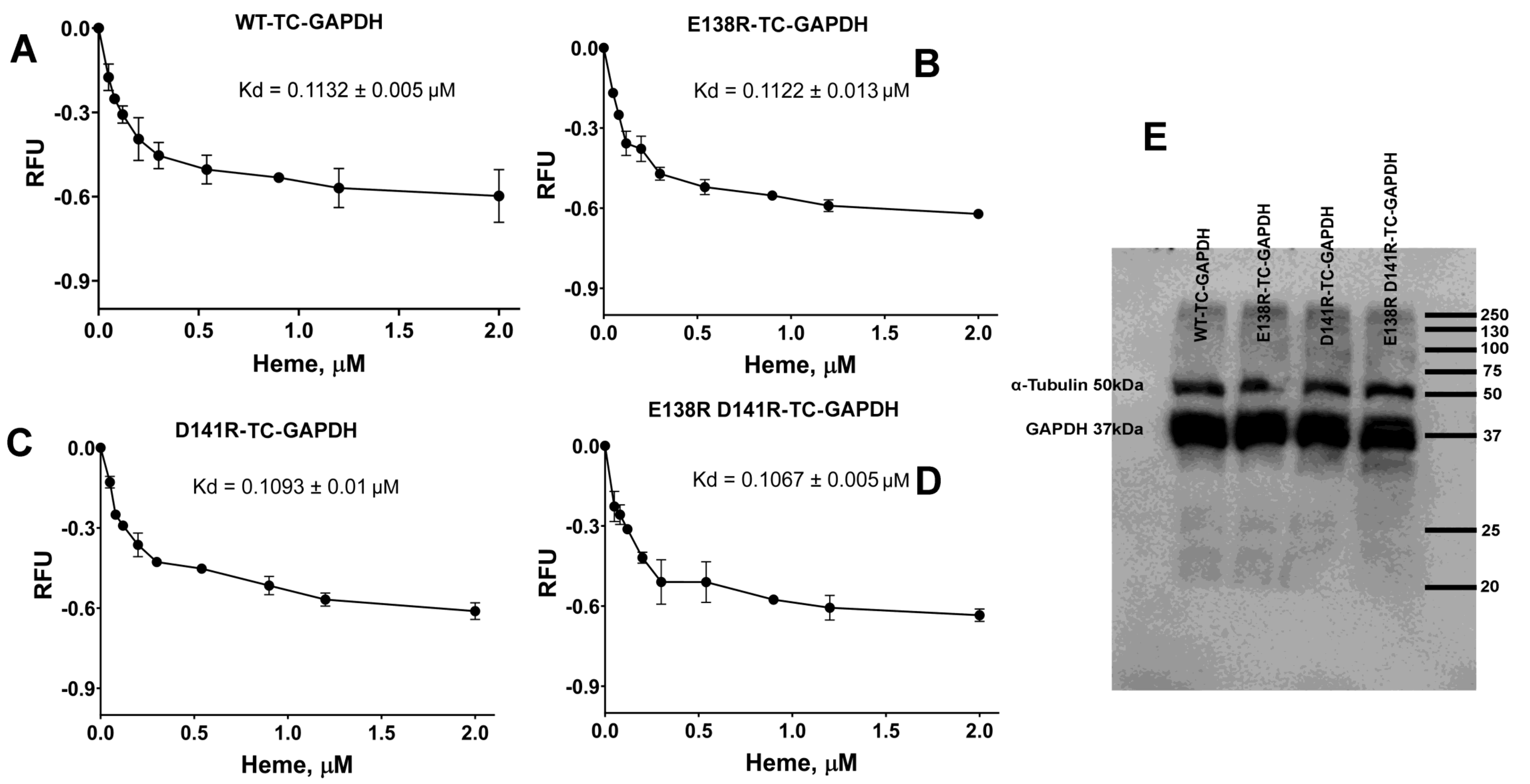

Fig. S9: Heme titrations of FlAsH-labeled WT-, E138R-, D141R- and E138R + D141R-TC-GAPDH in HEK293T cell supernatants. (A-D) Cell supernatants expressing each indicated FlAsH-labeled TC-GAPDH protein were titrated with various doses of hemin chloride at RT and the resulting quenching of FlAsH fluorescence was measured. (E) Protein expressions of the various TC-GAPDH constructs in the HEK293T cell supernatants. Data is shown as mean ± s.d., n=3.

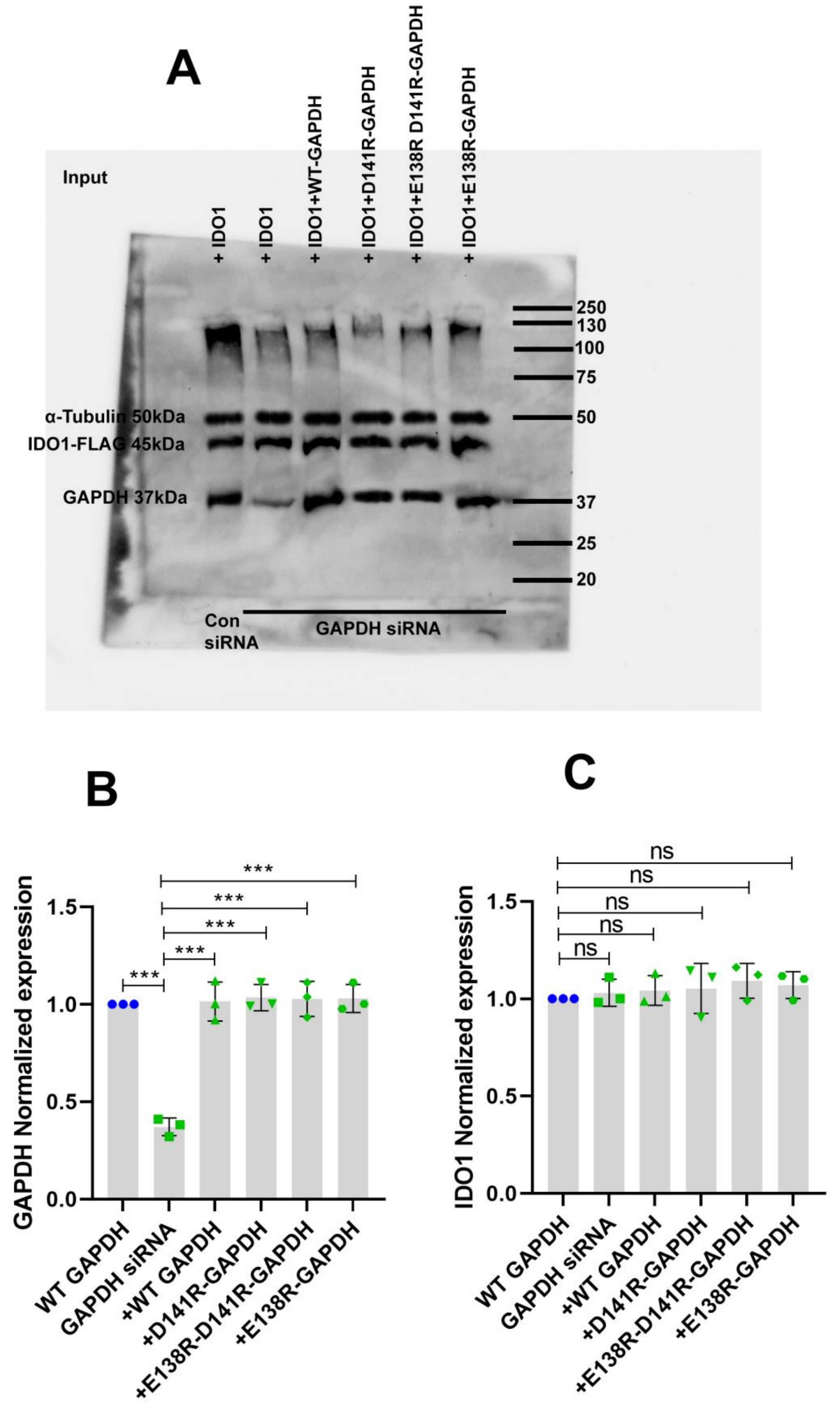

Fig. S10: Interaction of WT-IDO1 with WT-, E138R-, D141R- and E138R D141R-TC-GAPDH in HEK293T cells treated with control siRNA (blue) and GAPDH siRNA (green). (A) Input blot showing the silencing of endogenous GAPDH and further rescue of the various GAPDH proteins from siRNA resistant plasmids. WT-IDO1 expressions are also shown in the same samples. (B) GAPDH expression was normalized to that of α-Tubulin using densitometry. (C) IDO1 expression was normalized to that of α-Tubulin using densitometry. Data is shown as mean ± s.d., n=3. ***p<0.001, ns, not significant.

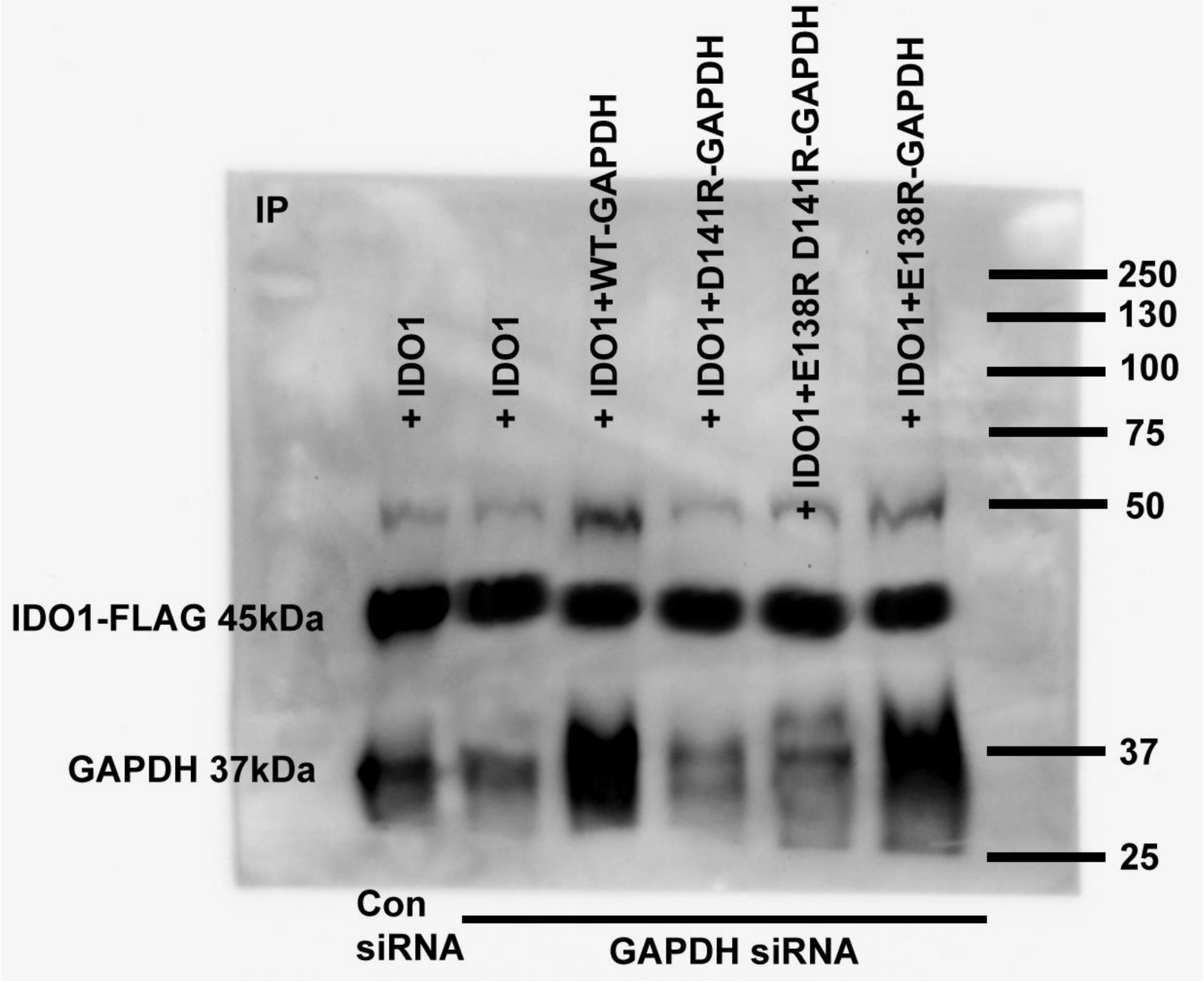

Fig. S11: Interaction of WT-IDO1 with WT-, E138R-, D141R- and E138R D141R-TC-GAPDH in HEK293T cells treated with control siRNA and GAPDH siRNA. Full IP blot showing levels of different GAPDH co-IP'd with IP'd IDO1-FLAG. The cropped image of this IP blot has been shown in Fig. 3A.

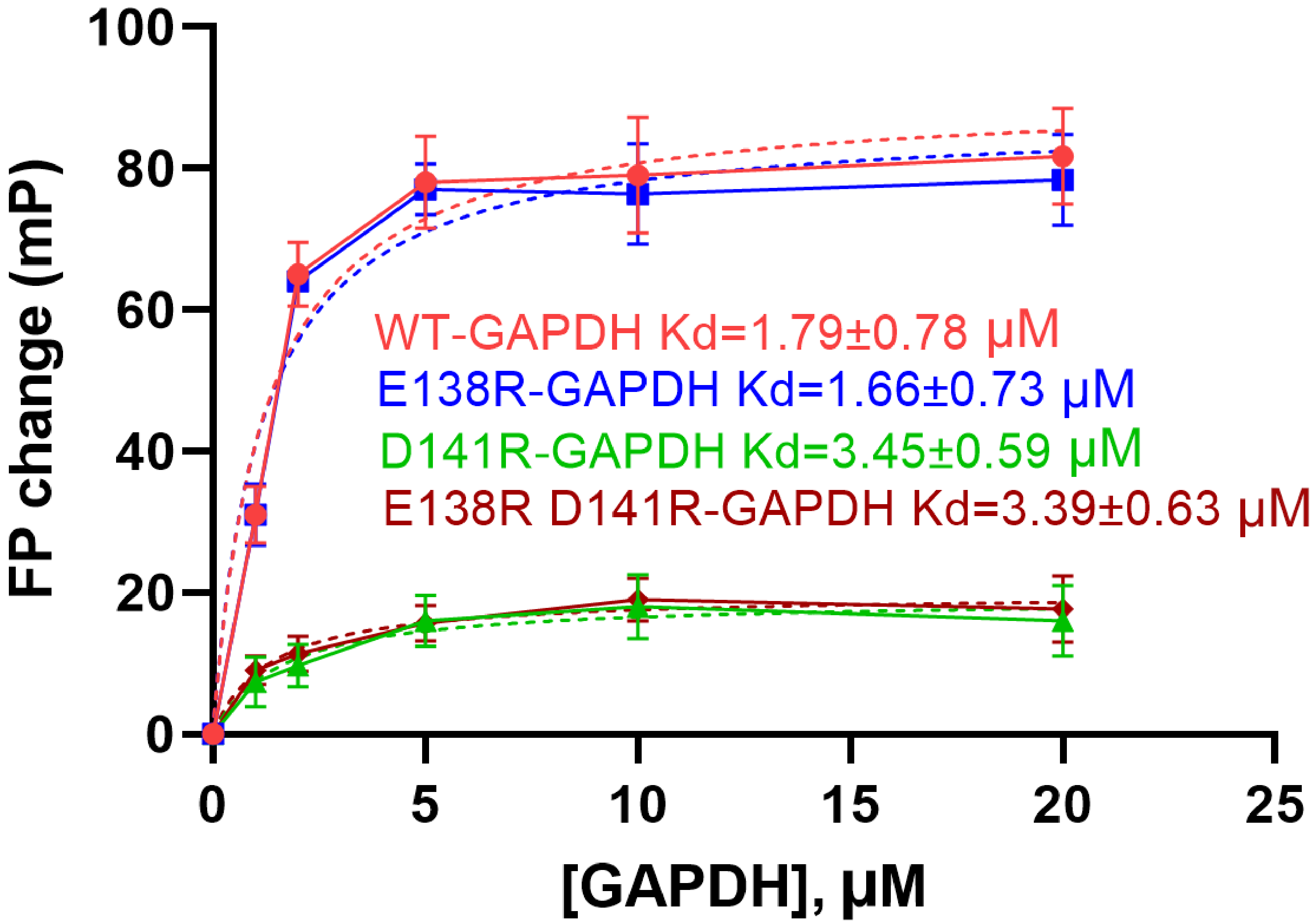

Fig. S12: Interaction between purified WT-IDO1 (100 nM) and various purified GAPDH proteins determined by the gain in residual fluorescence polarization. Different doses of WT-, E138R-, D141R- and E138R D141R-TC-GAPDH proteins were titrated against 100 nM of AF488 labeled WT-IDO1. Data is shown as mean ± s.d., n=3.

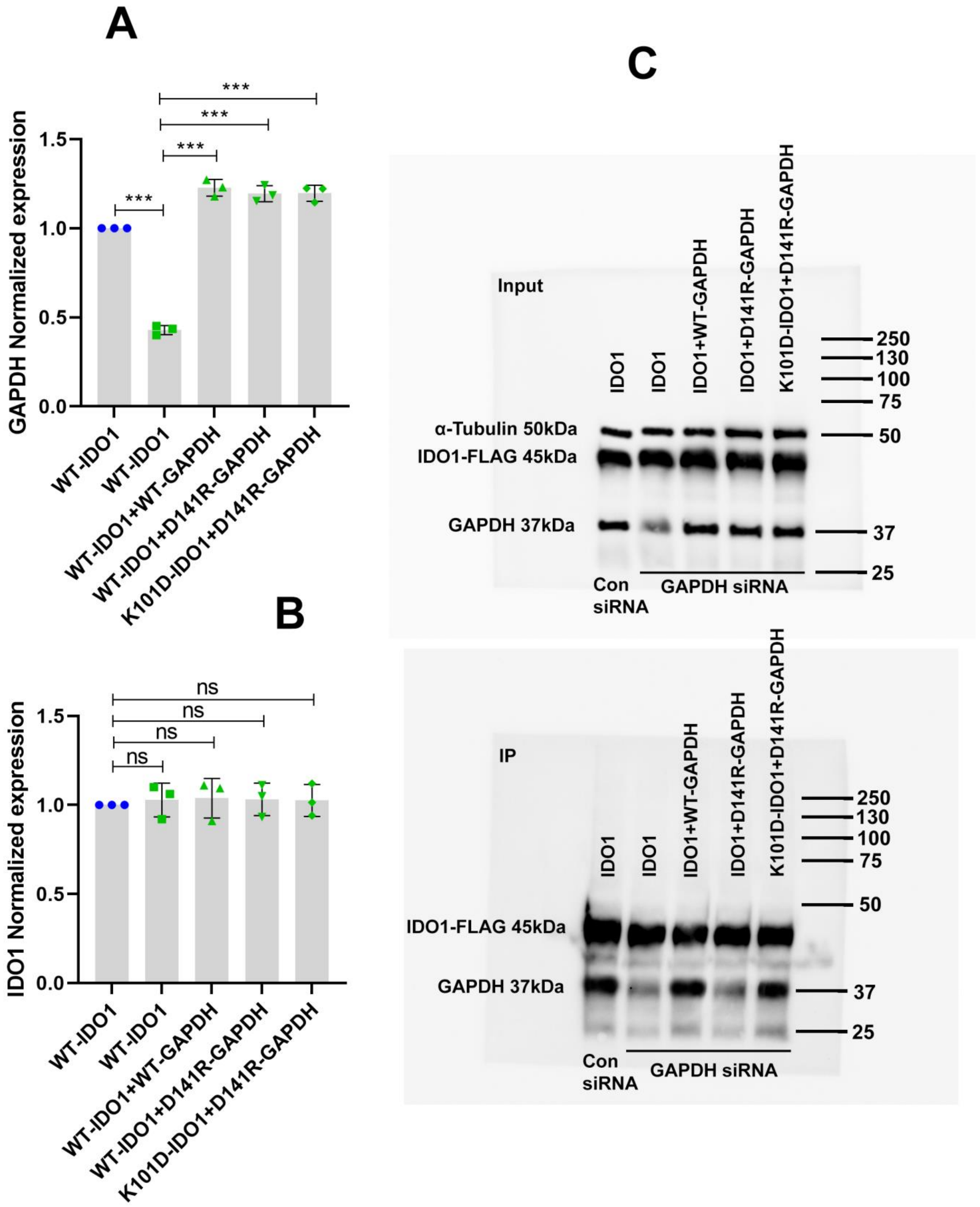

Fig. S13: Charge reversal experiment between IDO1 and GAPDH to rescue their interaction. We used D141R-GAPDH and K101D-IDO1 to complement the charges in HEK293T cells treated with control siRNA (blue) and GAPDH siRNA (green). (A) GAPDH expression was normalized to that of α-Tubulin using densitometry. (B) IDO1 expression was normalized to that of α-Tubulin using densitometry. (C) Full IP blot showing levels of different GAPDH co-IP'd with IP'd IDO1-FLAG (lower). Cropped image of this IP blot has been shown in Fig. 4A. Full input blot shows the protein expression levels of the IDO1 proteins and the silencing of endogenous GAPDH and expression of different GAPDH from siRNA resistant plasmids (upper). Data is shown as mean ± s.d., n=3. ***p<0.001, ns, not significant.

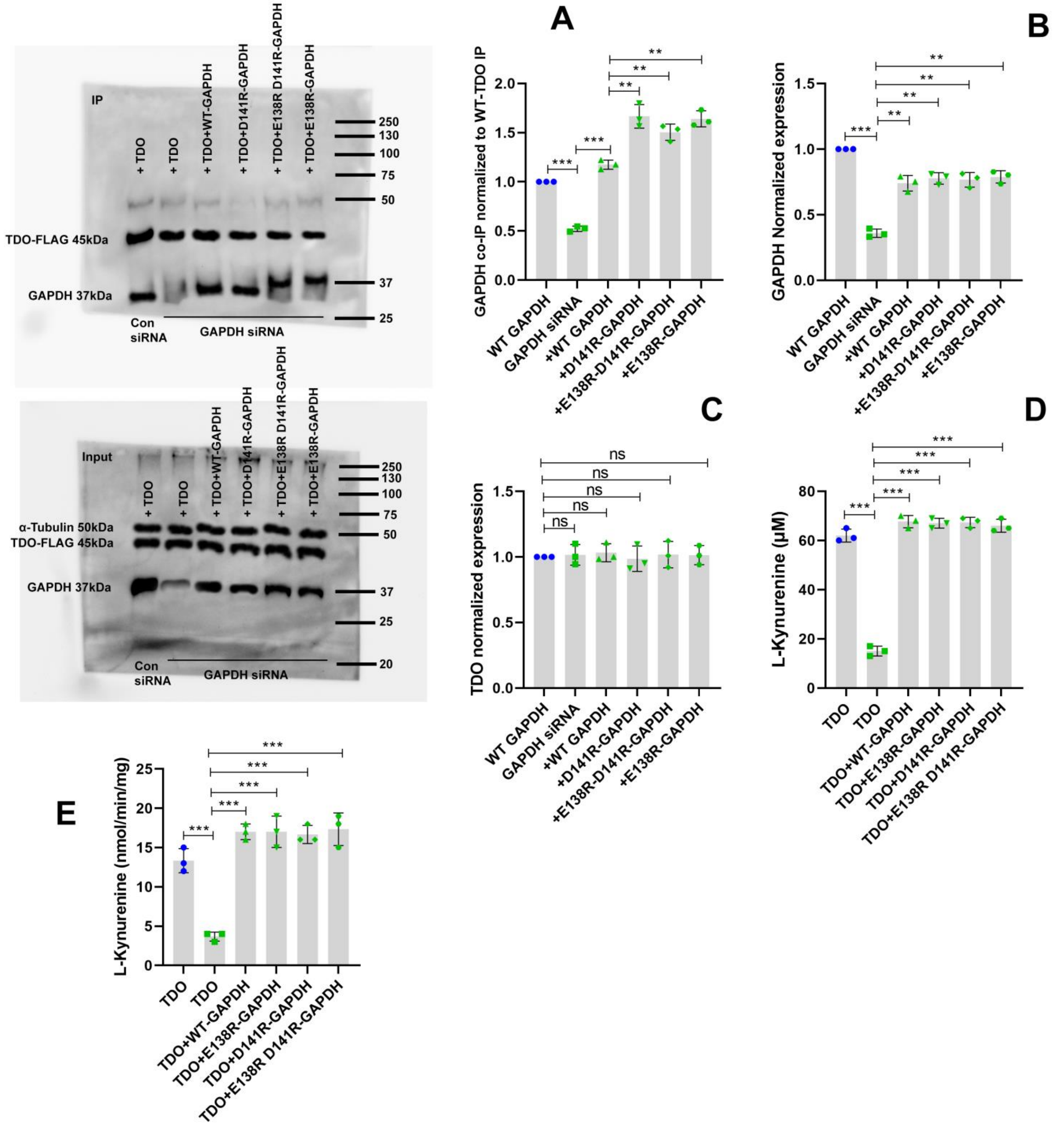

Fig. S14: Testing the WT-, E138R-, D141R- and E138R D141R-TC-GAPDH in HEK293T cells treated with control siRNA (blue) and GAPDH siRNA (green) in their ability to activate WT-TDO. (A) Interaction of WT-TDO with WT-, E138R-, D141R- and E138R D141R-TC-GAPDH. IP blot showing levels of different GAPDH co-IP'd with IP'd TDO-FLAG. Input blot showing the silencing of endogenous GAPDH and further rescue of the various GAPDH proteins from siRNA resistant plasmids. WT-TDO expressions are also shown in the same samples. GAPDH co-IP'd with IP'd TDO-FLAG was normalized to that IP'd TDO using densitometry. (B) GAPDH expression was normalized to that of α-Tubulin using densitometry. (C) TDO expression was normalized to that of α-Tubulin using densitometry. (D) Activity of WT-TDO in medium of HEK293T cells treated with control siRNA (blue) and GAPDH siRNA (green) and rescued with WT-, E138R-, D141R- and E138R D141R-TC-GAPDH. (E) Activity of WT-TDO in supernatant of HEK293T cells under the same experimental conditions. Data is shown as mean ± s.d., n=3. $^{**}p<0.01$, $^{***}p<0.001$, ns, not significant.

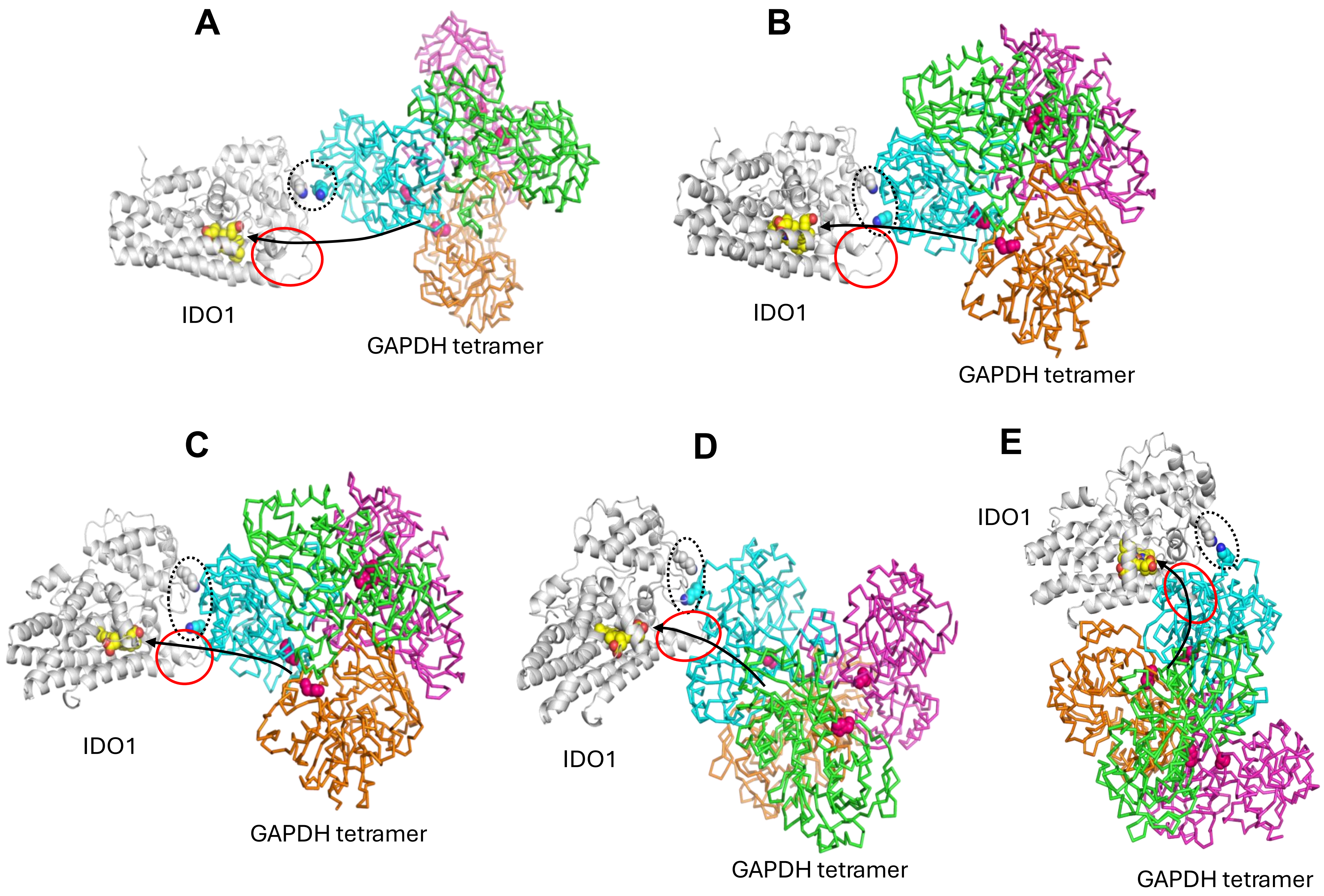

Fig. S15. Additional GAPDH-IDO1 model complexes that were computer-generated using the available protein crystal structures along with ColabFold and HADDOCK software. The IDO1 (gray) and GAPDH tetramer (multi-colored) proteins are shown primarily as ribbon structures with some highlighted regions or residues shown in space filling designation. Highlights: IDO1 heme cofactor (yellow with red propionate carboxylates), Crosslinked Lys residues (space filling residues in dashed black ovals), IDO1 J-K loop (red ovals), GAPDH heme-binding His residues (pink balls), and possible heme transfer pathways (black arrows).

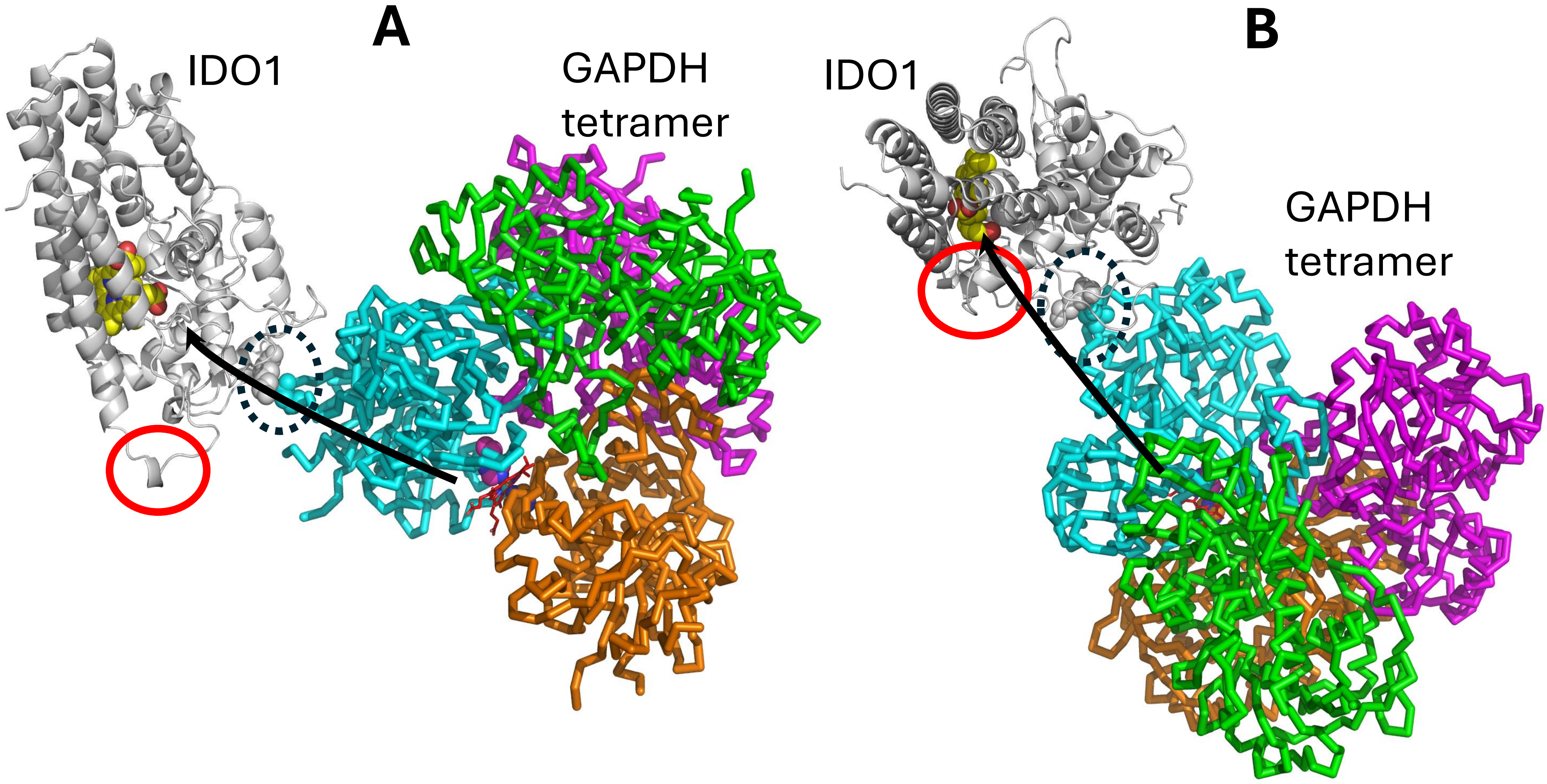

Fig. S16. Additional GAPDH-IDO1 model complexes that were computer-generated using the available protein crystal structures and Chai-1 software. The IDO1 (gray) and GAPDH tetramer (multi-colored) proteins are shown primarily as ribbon structures with some highlighted regions or residues shown in space filling designation. Highlights: IDO1 heme cofactor (yellow with red propionate carboxylates), Crosslinked Lys residues (space filling residues in dashed black ovals), IDO1 J-K loop (red ovals), GAPDH heme cofactor (red stick figure) and heme-binding His residue (purple balls), and possible heme transfer pathways (black arrows).
